# A pH-responsive metal-organic framework for the co-delivery of HIF-2α siRNA and curcumin for enhanced therapy of osteoarthritis

**DOI:** 10.1186/s12951-022-01758-2

**Published:** 2023-01-17

**Authors:** Zi-Jian Zhang, Ying-Ke Hou, Ming-Wa Chen, Xue-Zhao Yu, Si-Yu Chen, Ya-Ru Yue, Xiong-Tian Guo, Jin-Xiang Chen, Quan Zhou

**Affiliations:** 1 Department of Medical Imaging, Third Affiliated Hospital of Southern Medical University (Academy of Orthopedics Guangdong Province), Southern Medical University, Guangzhou, 510630 Guangdong People’s Republic of China; 2 NMPA Key Laboratory for Research and Evaluation of Drug Metabolism, Guangdong Provincial Key Laboratory of New Drug Screening, School of Pharmaceutical Sciences, Southern Medical University, Guangzhou, 510515 People’s Republic of China

**Keywords:** Osteoarthritis therapy, Metal-organic frameworks, Curcumin, Gene therapy, pH-Responsive materials

## Abstract

The occurrence of osteoarthritis (OA) is highly correlated with the reduction of joint lubrication performance, in which persistent excessive inflammation and irreversible destruction of cartilage dominate the mechanism. The inadequate response to monotherapy methods, suboptimal efficacy caused by undesirable bioavailability, short retention, and lack of stimulus-responsiveness, are few unresolved issues. Herein, we report a pH-responsive metal-organic framework (MOF), namely, MIL-101-NH_2_, for the co-delivery of anti-inflammatory drug curcumin (CCM) and small interfering RNA (siRNA) for hypoxia inducible factor (HIF-2α). CCM and siRNA were loaded via encapsulation and surface coordination ability of MIL-101-NH_2_. Our vitro tests showed that MIL-101-NH_2_ protected siRNA from nuclease degradation by lysosomal escape. The pH-responsive MIL-101-NH_2_ gradually collapsed in an acidic OA microenvironment to release the CCM payloads to down-regulate the level of pro-inflammatory cytokines, and to release the siRNA payloads to cleave the target HIF-2α mRNA for gene-silencing therapy, ultimately exhibiting the synergetic therapeutic efficacy by silencing HIF-2α genes accompanied by inhibiting the inflammation response and cartilage degeneration of OA. The hybrid material reported herein exhibited promising potential performance for OA therapy as supported by both in vitro and in vivo studies and may offer an efficacious therapeutic strategy for OA utilizing MOFs as host materials.

**Supplementary Information:**

The online version contains supplementary material available at 10.1186/s12951-022-01758-2.

## Introduction

The degenerative disease-osteoarthritis (OA) is a prevalent health issue that causes joint dysfunction and chronic disability worldwide [1, 2]. Though various biochemical factors contribute to the development of OA, its underlying mechanisms remain unclear [3–5]. Typically, this disease is characterized by a greatly reduced lubrication, causing cartilage damage and successive joint inflammations [6, 7]. Specifically, the continuous inflammatory response and excessive matrix metalloproteinases (MMPs) stimulated by up-regulated inflammatory cytokines degrade the extracellular matrix (ECM) and reduce chondrocyte activity. As a result of this event, chondrocyte catabolism is activated and chondrocyte proliferation is inhibited, resulting in cartilage degradation and damage [8, 9].

Current treatments of OA are primarily based on the oral administration of nonsteroidal anti-inflammatory drugs (NSAIDs), intra-articular (IA) injection of steroid drugs, and surgery for patients with severe conditions [10, 11]. Long-term use of these treatments has detrimental effects on the gastrointestinal and cardiovascular systems [12]. Curcumin (CCM), a natural polyphenol, has effective anti-inflammatory and antioxidant properties [13]. With minimal acute or chronic toxicity, CCM can be administered for long periods and has garnered massive attention from medical researchers for the treatment of inflammation-related diseases [14, 15]. A previous study demonstrated that CCM suppressed pro-inflammatory cytokines and inflammasome-activation by inhibiting NF-κB pathway [16]. Moreover, CCM indirectly inhibited the expression of MMPs in OA-affected chondrocytes preventing matrix degradation and inflammation [13]. Regrettably, the poor hydrophilicity and absorption of CCM contribute to its limited bioavailability [17]. Most importantly, owing to the complex inflammatory response and metabolic disorders in OA-affected sites, single-modal therapies rarely provide the intended therapeutic efficacy [5, 18]. Therefore, multi-modal therapy is an efficacious approach to achieving optimal therapeutic effects.

Gene therapy has been studied for various diseases recently, such as cancer, neurological disorders, and other genetic diseases [19]. In particular, RNA interference holds great promise for the treatment of OA owing to its high specificity in targeting and suppressing the expression of its complementary gene messenger RNA (mRNA) [20, 21]. It’s well known that HIF-2α plays a catabolic role in OA. The increased HIF-2α mediates the response of chondrocytes to hypoxia, as well as the expression of catabolic factors in hypoxic synovium during OA pathogenesis. It also stimulates the expression of cytokines, chemokines, and MMPs [22–24]. Since HIF-2α has been demonstrated as a crucial pathogenic target during the progression of OA, the down-regulation of HIF-2α expression in local inflammatory joints might be a promising approach to alleviating cartilage degeneration [25, 26]. Nevertheless, gene therapy limited clinical application because of easy enzymatic degradation, low transfection efficiency, unspecific biodistribution, and uncontrolled release of siRNA, thereby posing a challenge in developing platforms for the delivery of siRNA to chondrocytes [27–29]. Previous studies reported that metal-organic frameworks (MOFs) have attracted extensive attention in the gene therapy field [30].

Drug delivery systems can provide drug-controlled release and prevent undesired premature leakage, reducing the dosing frequencies of medication application. Importantly, controlled drug release systems, upon exposure to inflammation tissues, not only prolonged drug release but also promoted specificity to tissue and cells [31]. Inflamed joints have weakly acidic environments during cartilage degradation, which provide a suitable trigger for the drugs released [32–34]. Based on the above characteristics, a pH-responsive platform for the co-delivery of various therapeutic agents needs to be developed [31, 35, 36]. MOFs are emerging porous coordination polymers, composed of metal ions and organic ligands connected through the coordination bond, which provides firmness and flexibility to the geometric framework [37–39]. MOFs possess several notable properties as a drug carrier, such as high drug loading capacity, effective pH-responsiveness and excellent biocompatibility and biodegradability [34, 40, 41]. MIL (Materials Institute Lavoisier) family is a type of MOF widely used owing to their easy synthesis, stability, porous characteristics and high biocompatibility [39, 42]. Previous studies reported, MIL-101-NH_2_ showed little toxicity even up to 400 μg/mL in HeLa cell lines [43] and up to 100 μg/mL in mouse embryonic fibroblasts [44]. Specifically for this study, the mesoporous(III) MIL-101-NH_2_ was selected as the host material as it could sustained-release the siRNA and anti-inflammatory drugs by virtue of its pH-responsiveness and biodegradability.

**Table 1:** The primer sequences used for qRT–PCR

Gene	Forward primer	Reverse primer
GAPDH	CTCAACTACATGGTCTACATGTTCCA	CTTCCCATTCTCAGCCTTGACT
Acan	TCCACATCAGAAGAGCCATAC	AGTCAAGGTCGCCAGAGG
Col2a1	CTTAGGACAGAGAGAGAAGG	ACTCTGGGTGGCAGAGTTTC
SOX9	CAGTCCCAGCGAACGCACAT	TGCTGCTGCTGCTCGCTGTA
MMP3	GGCTGTGTGCTCATCCTACC	TGGAAAGGTACTGAAGCCACC
MMP13	AAAGAACATGGTGACTTCTACC	ACTGGATTCCTTGAACGTC
HIF-2α	CTGAGGAAGGAGAAATCCCGT	TGTGTCCGAAGGAAGCTGATG
IL-6	AGTCCTTCCTACCCCAATTTCC	TTGGTCCTTAGCCACTCCTTC
Adamts-5	TGTGGTGCGCCAAGGCCAAA	CCCTGTGCAGTAGCGGCCAC
COX-2	GATGACGAGCGACTGTTCCA	CAATGTTGAAGGTGTCCGGC

Additionally, oral medications always have a low absorption rate owing to the deficiency of blood vessels in articular cartilage [45, 46]. Since the reduction of exposure and long retention at the OA joint [47, 48], local IA injection is a promising treatment option for OA with minimal extra-articular symptoms [49–51]. Hyaluronic acid (HA) is a non-sulfated, naturally occurring nonprotein glycosaminoglycan, regarded as one of the best biopolymers for its high biocompatibility and biodegradability [52, 53]. Therefore, HA was selected to premix nanoparticles with the aim of improving the dispersibility and hydrophilicity of nanoparticles as well as the lubrication of cartilage.

**Scheme 1:**
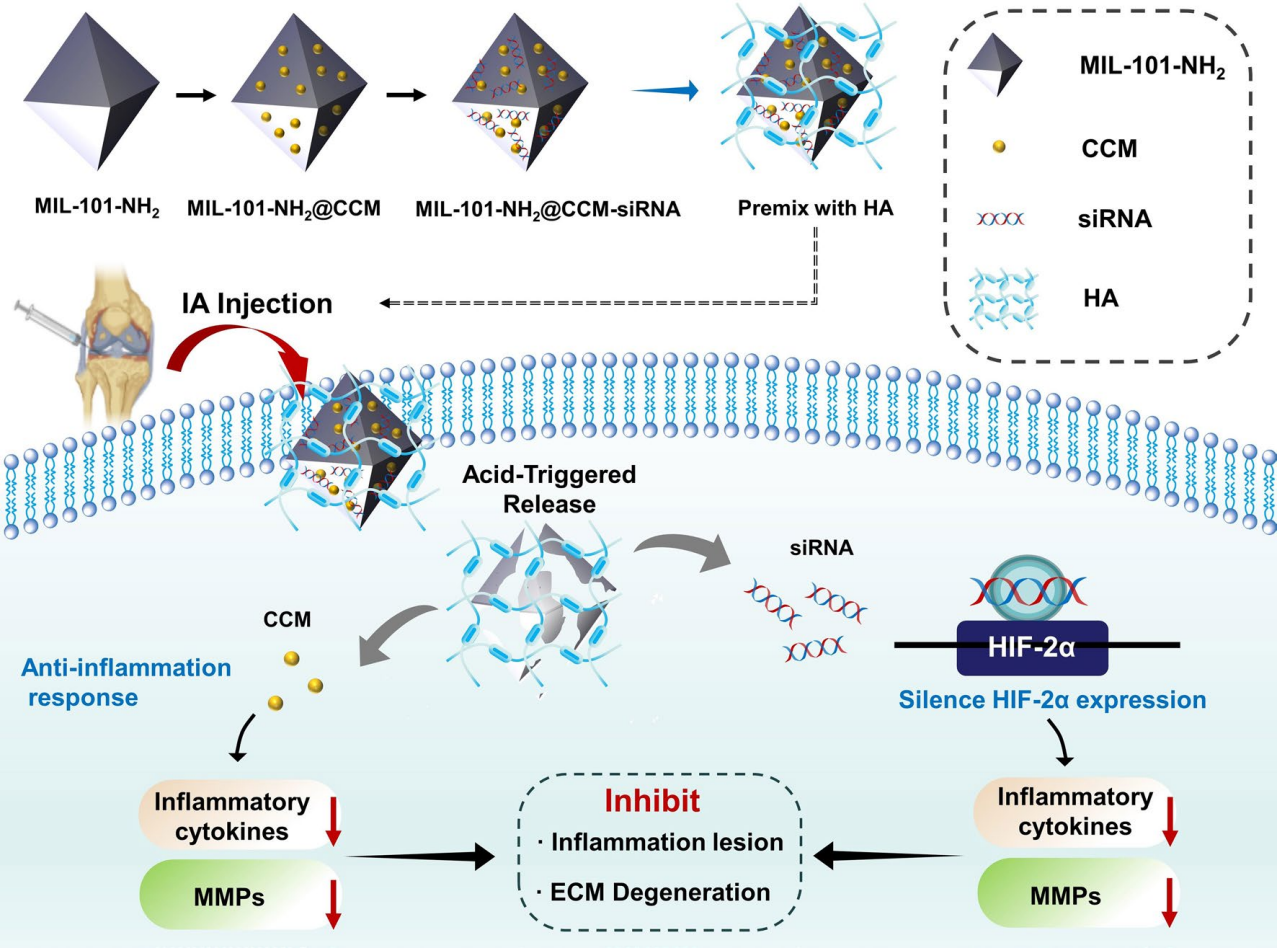
Preparation procedure of MIL-101-NH_2_@CCM-siRNA nanoparticles and schematic illustration of MIL-101-NH_2_@CCM-siRNA nanoparticles (premixed with the HA solution) for OA therapy

In this study, an injectable drug codelivery system (designated as MIL-101-NH_2_@CCM-siRNA) was constructed by loading CCM and anti-HIF-2α siRNA (siHIF-2α) within the MIL-101-NH_2_ to synergistically alleviate OA progression (Scheme 1). This hybrid nanocomposite shows the following features: (i) the pH-sensitive MIL-101-NH_2_ could promise a controlled and sustained release of CCM in OA-affected sites; (ii) the localized and sustained delivery of siHIF-2α from nanoparticles could result in significant HIF-2α silencing via the prevention of nuclease cleavage; (iii) the application of CCM and siHIF-2α could further synergistically inhibit the hypoxia-induced chondral dysfunction; and (iv) the IA injection could alleviate allergic symptoms or systemic toxicities to ensure good biocompatibility.

## Materials and methods

### Materials and reagents

Iron chloride hexahydrate (FeCl_3_⋅6H_2_O, 99.96%) and CCM were purchased from Shanghai Macklin Biochemical Co., Ltd (China). 2-Amino terephthalic acid (BDC-NH_2_, 99%), N,N-dimethylformamide (DMF) and Hyaluronic acid (HA, MW: 4.0 × 10^5^ Da) were obtained from Shanghai Aladdin Biochemical Co., Ltd (China). All siRNA duplexes were synthesized by Sangon Biotech Co., Ltd (China). The sequence of mice siHIF-2α: sense: 5′-CGU-GAG-AAC-CUG-AGU-CUC-A-3′, antisense: 5′-UGA-GAC-UCA-GGU-UCU-CAC-G-3′; siHIF-2α labeled with Cy5 was abbreviated as siCy5. DEPC-treated water, reactive oxygen species (ROS) Assay Kit and LysoTracker Green and MitoTracker Green were obtained from Beyotime Institute of Biotechnology (China). Recombinant Murine IL-1beta (IL-1β) was purchased from PeproTech Inc (China). The methyl-thiazolyl bromide (MTT) and 4′,6-diamidino-2-phenylindole (DAPI) and 2′,7′-dichlorofluorescin diacetate (DCFH-DA) were obtained from Zhengzhou Acme Chemical Co., Ltd. The antibody of matrix metalloproteinase13 (MMP13) was purchased from Abcam (China). ChamQ SYBR qPCR Master Mix, HiScript^®^ III-RT SuperMix for qRT–PCR (qRT–PCR = quantitative real-time polymerase chain reaction) (+ gDNA wiper) were purchased from Nanjing Vazyme Biotech Co., Ltd (China). Primes for Acan, Col2a1, SOX9, Interleukin-6 (IL-6), MMP13, HIF-2α, MMP3, thrombospondin motifs-5 (Adamts-5), cyclooxygenase-2 (COX-2), and housekeeping gene of GAPDH were purchased from Tsingke Biological Technology Co., Ltd (China). Collagenase II, Hematoxylin&Eosin stain Kit, Toluidine bule Stain Kit, modified Safranin O/ fast Green Stain Kit were purchased from Solarbio Science & Technology Co., Ltd (China). All reagents and solvents were commercially available and used as received.

### Apparatus

The morphological analysis of nanoparticles was performed on Hitachi HT7700 transmission electron microscope (TEM) and Hitachi S-4700 field-emission scanning electron microscope (SEM). Dynamic light scattering (DLS) and surface charge (zeta potential) of the different nanoparticles were analyzed using a Malvern Zetasizer Nano ZS (Malvern, England). qRT–PCR was conducted using LightCycler 96 system (Roche, Switzerland). Wide-angle powder X-ray diffraction (PXRD) patterns were conducted with the crystal phase of nanoparticles via D8-advance X-ray diffraction (Rigaku, Japan) using Cu Kα radiation over the 2θ range 5 − 30°. The Ultraviolet–visible (UV–vis) absorption spectroscopy was measured using a UV spectrophotometer (Lambda 25, Perkin Elmer, USA). Microscopy images of cells were obtained on Axio Observer A1 fluorescence inverted microscope and confocal laser scanning microscopy FV3000 (CLSM, Olympus Corporation, Japan). The reconstruction of micro-CT three-dimensional images were obtained on NanoScan1172.

### Preparation of CCM-loaded MIL-101-NH_2_

The MIL-101-NH_2_ was synthesized according to previously reported methods [43, 54]. Typically, the powder of FeCl_3_·6H_2_O (67.6 mg, 0.25 mmol) and BDC-NH_2_ (45.3 mg, 0.25 mmol) were independently dissolved in 10.0 mL DMF to form clear solutions. Then, the two solutions were mixed under ultrasonic wave for 10 min, and the mixture solution was left undisturbed for 12 h at 120 °C. The precipitated brownish-red product was obtained by centrifugation at 8000 rpm for 5 min, washed with DMF and ethanol three times, and then dried at room temperature overnight under vacuum to completely remove the residual solvent.

CCM-loaded MIL-101-NH_2_ nanoparticles (denoted as MC NPs) were prepared using the impregnation approach [43]. First, the MIL-101-NH_2_ solution was prepared by ultrasonic dispersion in ethanol. Next, CCM was dissolved in ethanol and homogeneously added into MIL-101-NH_2_ solution (1 mg/mL; 5 mL) with different weight ratios (Additional file 1: Table S1) of MIL-101-NH_2_/CCM, and finally stirred at a speed of 200 rpm for 24 h. After that, the solution was centrifuged at 8000 rpm for 5 min and washed with ethanol several times to remove the redundant and surface-absorbed CCM and obtain the final product, namely, MC NPs. Finally, the MC NPs were dried under vacuum overnight at room temperature prior to further application. Based on the standard curve, the CCM content of MC NPs was determined using a UV spectrophotometer, and the drug loading capacity (DLC) and drug loading efficiency (DLE) were calculated using Additional file 1: Equation S1 and S2.

### Preparation of siHIF-2α-loaded MIL-101-NH_2_@CCM complex

The MC NPs were filtered through a millipore filter (pore size: 0.22 µm) and were then dissolved in DEPC-treated water. Different volumes of MC NPs solutions were added dropwise into the siHIF-2α solution, and the weight ratios of MC NPs to siHIF-2α were varied from 5:1 to 35:1. Afterward, the mixtures were adjusted to the same final volume in DEPC-treated water, and the final concentration of siHIF-2α was 100 nM. Subsequently, the mixed solutions were incubated for 30 min with a shaking speed of 100 rpm at room temperature. The siHIF-2α-loaded MC nanoparticles-MIL-101-NH_2_@CCM-siRNA (denoted as MCS NPs) were centrifuged at 13,000 rpm for 15 min, washed thrice with DEPC-treated water. Then, the supernatant was discarded to remove the free siHIF-2α, and the precipitate of MCS NPs was finally collected and re-suspended in an aqueous solution at 4 ℃ for subsequent evaluations. As shown in Additional file 1: Table S2, siHIF-2α and Cy5-labeled siHIF-2 (siCy5) were used in different experiments. And Additional file 1: Table S3 presents the weight ratio of MC to siHIF-2α in MCS NPs.

### siHIF-2α loading affinity of MC NPs assay

The siHIF***-***2α binding affinity of MC NPs was confirmed by fluorescence spectrum and gel electrophoresis. First, the binding affinity of siHIF-2α was preliminarily evaluated via fluorescence spectrum with the excitation and emission wavelengths of siCy5 being 650 nm and 670 nm, respectively. Then, the concentration of the siCy5 solution was fixed to 100 nM. With the addition and agitation of MIL-101-NH_2_, MC NPs at a certain concentration, the emission fluorescence peak of the measured mixed solution gradually decreased in different degrees, which was confirmed by the fluorescence spectrum.

The ability to absorb siHIF-2α of MC NPs was also confirmed by gel retardation assay. The binding affinity of MC NPs with different DLCs was evaluated, compared with the naked siHIF-2α and MIL-101-NH_2_-siRNA NPs (denoted as MS NPs). First, 20 μg of samples (MC NPs with different DLC) were incubated with 100 nM siHIF-2α aqueous solution. The obtained solutions were centrifuged, the precipitations of MCS NPs were resuspended in 10 μL DEPC-treated water, and the supernatants were collected for comparison. The samples were loaded onto the preheated 4% agarose gel by using a previous reported method [55]. Then, the electrophoresis experiment was performed at 180 V for 20 min, and the gel was stained with ethidium bromide solution for 30 min. Ultimately, the UV imaging system was used to investigate the loading capacity of these samples.

### In vitro CCM and siHIF-2α release studies

To determine the drug release profile, we suspended 4 mg of MC NPs in 40 mL of phosphate-buffered saline (PBS) + 1% v/v Tween 80 and shook the same at 120 rpm for 40 min [43]. Tween 80 was added to enhance the stability and solubility of CCM in PBS. At a predetermined time, 1 mL of the solution was removed for evaluation, and replenished with 1 mL of fresh PBS + 1% v/v Tween 80 to maintain the constant condition. The withdrawn solution was analyzed with a UV–Vis spectrophotometer to obtain the concentration of the released drug based on the standard curve of CCM in PBS + 1% v/v Tween 80. Afterward, a time-dependent drug release percentage was calculated with three repeated experiments. The standard curve of CCM in PBS (1% *v*/*v* Tween 80; pH = 5.0, pH = 6.5, and pH = 7.4) and the equations for the calculation of the percent of release drugs are presented in the additional files.

To evaluate the release rate of siHIF-2α, the MCS NPs (1.0 mg/mL) were dispersed in PBS with various pH values (5.0, 6.5 and 7.4). At a predetermined time, 100 μL of the above solutions was collected and centrifuged at 13,000 rpm three times. The supernatant was then collected for measuring absorbance at 260 nm. All release experiments were conducted in triplicate. The cumulative release of siHIF-2α was calculated by dividing the released amount by the total encapsulated amount.

### Stability

The stability of MCS NPs was analyzed by observing the nanoparticle-diameters. First, 1 mg of MCS NPs was dispersed in 10 mL PBS, DMEM/F12, and FBS (pH = 7.4) at room temperature for 14 days, respectively. At indicated time points, the DLS of the MCS NPs solution was observed by Malvern Zetasizer Nano ZS. And the residues were collected for further PXRD measurement.

### Isolation and culture of primary chondrocytes from mice

Primary articular chondrocytes were harvested from one-week-old C57BL/6 mice, which were sacrificed and immersed in 75% alcohol to for 10 min to disinfection. Under aseptic conditions, the total knee cartilage of exposed mice was harvested with sterile equipment and digested with 0.25% trypsin for 30 min. The residual cartilage tissue was further separated under a microscope, and the further subdivided cartilage tissue was digested with type II collagenase for 6 h. Following this, the harvested chondrocytes were filtered with a 70 μm cell strainer and then rinsed using sterile PBS. The resultant mice primary chondrocytes were cultured in DMEM/F12 with 10% fetal bovine serum (FBS) and 1% penicillin and streptomycin. All the chondrocytes were cultured at 37 ℃ and 5% CO_2_ and passaged at nearly 80% confluency until the second or third for further use to avoid changes in phenotype.

### MTT assay

By using the MTT assay, we investigated the influences of CCM, MIL-101-NH_2_, MC NPs, and MCS NPs on the proliferation of chondrocytes. The chondrocytes were seeded in 96-well plates (8000 cells per well) and incubated overnight. The medium was removed and replaced by 100 μL of fresh culture medium (10% FBS in DMEM/F12 medium). Various concentrations of CCM, MIL-101-NH_2_, MC NPs and MCS NPs were added into the culture medium separately. In the following 24 h incubation, 100 μL/well MTT solution (0.5 mg/mL) was added and further incubated for 4 h at room temperature. Finally, the supernatants were discarded, and the formazan crystals were dissolved in DMSO (150 μL/well) with the absorbance measured by a multiple microplate reader at 490 nm.

### Live/dead cell staining

To measure the cell biocompatibility of MCS NPs, live and dead cell staining using calcium fluorescein AM (green fluorescence) and propidium iodide (PI, red fluorescence) were performed, respectively. Chondrocytes at 80% confluency in 24-well plates were incubated with concentrations of 100 μg/mL MCS NPs for 24, 48 and 72 h. Then, the chondrocytes were washed with PBS before using the inverted fluorescence microscopy. As per the manufacturer’s instructions, green fluorescence indicates viable cells with esterase activity, whereas red fluorescence shows dead cells.

### Intracellular reactive oxygen species (ROS) measurement

To detect the intracellular reactive oxygen species (ROS) generation induced by MIL-101-NH_2_, an ROS assay kit was applied. Three groups of chondrocytes were studied and compared: (1) Control group, with chondrocytes incubated with culture medium only; (2) Rosup group, with chondrocytes incubated with culture medium and stimulated by 1 μL of 50 mg/mL active oxygen (Rosup) for 30 min as comparison; and (3) MIL-101-NH_2_ group, with chondrocytes incubated with culture medium and 100 mg/mL MIL-101-NH_2_ for 24 h. Then, after washing with a serum-free medium, chondrocytes were stained with DCFH-DA. After incubation of each group with 10 μM of DCFH-DA for 30 min, the chondrocytes were washed thrice with PBS. Subsequently, the fluorescence intensity was determined via fluorescence microscopy and ROS levels were evaluated by ImageJ.

### Cellular uptake studies of MCS NPs

To initiate the processes of CCM internalization and transfection and gene silencing, MCS NPs were required to first cross cell membranes and be uptaken by chondrocytes. Owing to the intrinsic green fluorescence of CCM and the red fluorescence of siCy5, the cellular uptake of MCS NPs was investigated using a confocal laser scanning microscope (CLSM). Therefore, the cellular uptake was demonstrated by two different intracellular fluorescence approaches. In this study, chondrocytes with a density of 10^5^ cells per dish, were seeded in dishes with glass bottom and incubated in the culture medium for 24 h. After this, the chondrocytes were treated with the MCS NPs (100 µg/mL) in fresh DMEM at different durations (0, 2, 4, 6, and 8 h). Finally, the medium was removed, and the cells were washed with PBS (pH = 7.4), and then stained with DAPI (10 µg/mL) for 20 min before being observed by a CLSM. Besides, to evaluate the internalization pathway of nanoparticles, Lysotracker Green and MitoTracker Green were incubated with MS NPs at a predetermined time. The co-localization rate was quantitatively determined using ImageJ based on the CLSM images.

### Lysosome escape

To visualize the co-localization of internalized MCS NPs within the lysosomal compartments, the chondrocytes were incubated with MIL-101-NH_2_ containing siCy5 for different durations at 37 °C. The chondrocytes were washed with PBS several times, fixed with 4% paraformaldehyde for 20 min at 4 °C, and sequentially stained with Lysotracker Green (100 nM) for 1 h, and with DAPI (10 µg/mL) for 20 min at room temperature before being observed under the CLSM. A time-dependent study of lysosome escape was performed by incubation MCS NPs with chondrocytes for 30, 60, 120, 240, and 360 min followed by Lysotracker Green and DAPI staining. Subsequently, the time-dependent co-localization of MCS NPs and lysosomes was observed by CLSM, and the co-localization efficiency was quantitatively analyzed using ImageJ based on the CLSM images.

### Treatment of inflammatory chondrocytes stimulated by IL-1β

The chondrocytes were divided into five groups: (1) Control group, with chondrocytes incubated with culture medium only; (2) IL-1β group, with chondrocytes incubated with culture medium and 10 ng/mL IL-1β for 24 h, simultaneously; (3) MC group, with chondrocytes incubated with culture medium, 100 μg/mL MC NPs and 10 ng/mL IL-1β for 24 h, simultaneously; (4) MS group, with chondrocytes incubated with culture medium,100 μg/mL MC NPs and 10 ng/mL IL-1β for 24 h, simultaneously; (5) MCS group: chondrocytes incubated culture medium with 100 μg/mL MC NPs and 10 ng/mL IL-1β for 24 h, simultaneously.

### Quantitative real-time polymerase chain reaction (qRT–PCR) assay

For RT–qPCR analysis, chondrocytes were seeded into six-well plates and incubation for 48 h. Then these chondrocytes were post-treated with MS NPs, MC NPs and MCS NPs followed by stimulating with 10 ng/mL IL-1β for 24 h. As previously reported, total RNA was extracted from chondrocytes using an RNA isolation kit [56]. One microgram of total RNA was used to reverse-transcribed and synthesize cDNA using a ReverTra Ace qPCR RT Kit.  Finally, the amplification of cDNA was performed by the SYBR Green real-time PCR Master Mix on a LightCycler 96 instrument. The specific primer sequences used in this study are listed in Table 1. 

### Safranin O staining in vitro

In the in vitro study, chondrocytes were fixed with 95% alcohol for 30 min in all groups. After washing with PBS and removing the residue of 95% alcohol, the chondrocytes were stained with safranin O dye for 10 min and then washed with PBS to remove the residual dye. Finally, five groups were measured using an inverted fluorescence microscope.

### Immunofluorescence staining

The prepared chondrocytes were fixed with 4% paraformaldehyde for 15 min, followed by permeabilization with 0.1% Triton X100 for 15 min. Subsequently, the chondrocytes were incubated with mouse anti-MMP13 antibodies (1:100 dilution) at 4 °C overnight. The chondrocytes were washed with PBS several times and then incubated with appropriate Alexa Fluor-coupled secondary antibodies for 1 h. The cell nuclei were stained with DAPI for 15 min. Ultimately, the images were obtained using an Olympus fluorescence microscope.

### Animal experiments

Animal experiments were approved by the Animal Ethics Committee of the Animal Experiment Center of Southern Medical University (Permit Number: 44002100029797). The C57BL/6 mice were obtained from the Animal Experiment Center of Southern Medical University. Animal experiments were conducted in accordance with the guidelines of the Animal Care Committee.

The model mice were randomly divided into five groups on average: the normal control group (NC group), destabilized medial meniscus (DMM) group, MC group, MS group, and MCS group (all premixed with the HA solution). Except for the NC group, the DMM surgery was applied to construct the OA model in male C57BL/6 mice (12 weeks old; n = 40). Anesthesia was used to expose the arthrosis, and the quadriceps was turned laterally to avoid damaging the patellar ligament after the arthrosis was exposed [57]. The medial meniscus was then dissected, and the medial meniscus ligament was transected in a way that did not damage the articular cartilage. Finally, the medial capsular incision was sutured after restoring the quadriceps well and closing the skin. Sham operations involving exposure of the joint capsule without MMTL transection were performed as a non-operated control group. Moreover, the mice in the MC, MS and MCS groups were intra-articular injected with 10 μL solution (1 mg/mL) into joints once per week for 4 weeks. The mice in these groups were sacrificed for analysis at weeks 4 and 8 after treatment. Besides, the body weights of mice were measured before these mice were sacrificed.

### Micro-CT scan

With the aid of a microtome imaging system, a micro-computed tomography (micro-CT) scan was performed on specimens corresponding to fixed knee joints. The small field was selected for scanning and correcting the CT value, with a 70 kV scanning voltage and 5 μm scan thickness. Three-dimensional knee reconstruction and image capture were performed using Mimics Medical software. After defining the interest region to encompass all osteophytes, the bone volume (BV) and articular space width were analyzed (relative to the NC group). Reconstructed and three-dimensional modeled data were obtained for further analysis.

### Histological staining in vivo

Knee joints from the mice were harvested, fixed in 4% paraformaldehyde for 48 h, and decalcified for 4–8 weeks in 10% ethylenediaminetetraacetate (EDTA). After serial dehydration, the joints were embedded in paraffin and sagittally sectioned at 4-μm thickness. These sections were dewaxed and stained with Hematoxylin&Eosin (H&E), safranin O/fast green and toluidine blue. Then, the sections were graded according to the scoring criteria reported by Osteoarthritis Research Society International (OARSI) [58].

Immunohistochemical staining was performed to analyze the secretion of MMP13. After dewaxed sections were washed with PBS, the sections were treated with 3% (v/v) hydrogen peroxide H_2_O_2_ for 15 min at room temperature to block endogenous peroxidase activity. After blocking with normal goat serum for 20 min at room temperature, primary MMP13 antibodies were added and incubated at 4 °C overnight. Afterward, the biotin-labeled horse radish peroxidase solution was added to the sections after 15 min of secondary antibody incubation. To develop colors and dye nuclei, 3, 3′-diaminobenzidine tetrahydrochloride was used as well as hematoxylin. Tissues sections were observed and photographed with an Olympus fluorescence microscope.

### Statistical analysis

All data were expressed as the mean ± standard deviation (SD), and all independent experiments were repeated at least three times. One-way analysis of variance (ANOVA) was used to assess group differences. *p < 0.05; **p < 0.01; ***p < 0.001 were considered statistically significant. Statistical analyses were performed by Origin 9 and GraphPad Prism 9.0.

## Results and discussion

### Synthesis and characterizations of MCS NPs

**Fig. 1:**
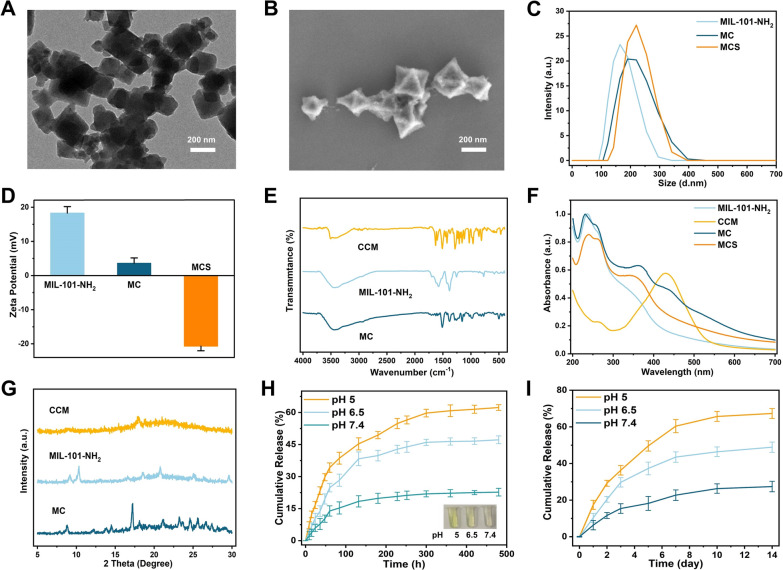
**A** TEM image of MIL-101-NH_2_@CCM-siRNA NPs. **B** SEM image of MIL-101-NH_2_@CCM-siRNA NPs. **C** Hydrodynamic diameter distribution and **D** Zeta potential of MIL-101-NH_2_, MC, and MCS NPs. **E** FT-IR spectra of CCM, activated MIL-101-NH_2,_ and MC NPs. **F** The UV–Vis absorption spectra of CCM, MIL-101-NH_2_, MC, and MCS NPs. **G** PXRD patterns of pure CCM, MIL-101-NH_2_, and MC NPs. **H** In vitro release of CCM from MIL-101-NH_2_@CCM in PBS-1% v/v Tween (pH = 5.0, 6.5, and 7.4). Inset: digital photographs of solutions**. I** In vitro release of siCy5 from MCS NPs in PBS (pH = 5.0, 6.5, and 7.4) at 37 ℃ ± 1 ℃

The MCS NPs were synthesized as detailed in the methods and characterized using multiple techniques. The TEM images (Additional file 1: Fig. S1A and Fig. 1A) showed that the diameters of MIL-101-NH_2_ and MCS NPs are around 200 nm. According to the SEM images (Additional file 1: Fig. S1B and Fig. 1B), both MIL-101-NH_2_ and MCS NPs exhibited regular octahedron structures, suggesting that the CCM encapsulation did not significantly affect the morphologies or size of MIL-101-NH_2_. Similarly, as displayed in Fig. 1C, DLS confirmed the size of MCS NPs to be around 200 nm, which is consistent with the TEM results, indicating that the MCS NPs are suitable for cellular and biological applications. The changes of the zeta potential in MIL-101-NH_2_, MC NPs and MCS NPs were also characterized (Fig. 1D). Because of the encapsulation of CCM molecules on MIL-101-NH_2_ NPs, the zeta potential of MC NPs decreased from positively charged (18.3 mV to 3.6 mV), and further reversed to negatively charged (− 20.7 mV) upon the conjunction of siHIF-2α.

The FT-IR spectra analysis of CCM, MIL-101-NH_2_, and MC NPs are shown in Fig. 1E. In the MC, a blue-shift from 3490 to 3426 cm^−1^ was observed in the stretching peak of the phenolic group, compared with the CCM. To further verify the successful encapsulation of CCM into the MIL-101-NH_2_, the UV–Vis spectrum was also investigated. As depicted in Fig. 1F, the MIL-101-NH_2_ had three absorption bands at 238, 335 and 375 nm, while the MC has a strong absorption band that appeared at 425 nm, which was close to the characteristic absorption peak of the CCM, further suggesting that CCM was already encapsulated into the framework of MIL-101- NH_2_. In the MSC, the absorbance peak was not significantly different from the absorbance peak in MC, because siHIF-2α absorbance locates at 260 nm, which is closed to 238 nm. The PXRD patterns of CCM, MIL-101-NH_2_ and MC were shown in Fig. 1G. Both CCM and MIL-101-NH_2_ patterns matched the PXRD pattern of MC, indicating that CCM loading had little impact on the spectrum of MIL-101-NH_2_. These results confirmed the successful synthesis and drug loading of MIL-101-NH_2_, and suggested that CCM encapsulation did not apparently affect the MIL-101-NH_2_ crystal structure. In conclusion, MIL-101-NH_2_ was successfully synthesized and loaded with CCM, and its crystal structure was not apparently affected by CCM encapsulation.

### CCM and siHIF-2α loading affinity in vitro

Based on the UV–Vis absorbance spectrum of CCM in ethanol with different concentrations (Additional file 1: Fig. S2A), the standard curve of CCM (Additional file 1: Fig. S2B) in ethanol is depicted herein by the characteristic absorbance at λ = 425 nm. The MC NPs with various DLCs and DLEs were obtained by incubating MIL-101-NH_2_ and CCM at different weight ratios for 24 h (Additional file 1: Table S1). As the weight ratios of MIL-101-NH_2_/CCM varied from 1/2 to 1/0.125, the DLC value of the MC NPs decreased from 42.5% to 25.9%. Meanwhile, the DLE value initially increased and then decreased, peaking at 69.5% when the weight ratio was 1/1. Several studies have shown that the interaction between the metal sites in MIL-101-NH_2_ and the hydroxyl group of CCM provided drug encapsulation with high DLC [59].

The gene vector loading capacity and transfection efficiency are heavily influenced by the binding affinity between gene vectors and siRNA. Therefore, to determine the siHIF-2α loading capacity of MC NPs, fluorescence spectrum and agarose gel electrophoresis were performed first. The siHIF-2α was loaded onto MC NPs by simply mixing an appropriate amount of siHIF-2α and MC NPs in DEPC-treated water to construct the final MCS NPs. The vacant Fe^3+^ sites on MIL-101-NH_2_ surfaces and the phosphate group of siHIF-2α provided the hydrogen bonds and multiple coordination bonds to form the nanoparticle-siRNA composites [39].

As shown in Additional file 1: Fig. S3A and S3B, the siHIF-2α binding capabilities of MC NPs were evaluated by the fluorescence spectrum. The fluorescent excitation/emission properties of siCy5) were examined, and the excitation and emission peaks at 650 and 670 nm, respectively, were observed. With the same concentration of siCy5, the characteristic emission peak declined sharper at the 25.9% DLC (CCM) group than the 42.5% DLC (CCM) after binding to MC NPs. The agarose gel retardation assay also showed the same consequence (Additional file 1: Fig. S4A and B). After binding to MC NPs, siRNA migration bands of MC faded differently depending on their DLC values, indicating that MC NPs can efficiently capture the free siRNA result from the vacant Fe^3+^ sites of MC NPs. However, compared with the MC NPs with 42.5% DLC (CCM), the band of 25.9% DLC (CCM) had a more efficient binding rate. In addition, the MC NPs with 25.9% DLC also had the highest DLE. On the basis of these results, MC NPs with DLC% of 25.9% and DLE% of 69.5% were selected for follow-up experiments.

### In vitro release profiles of CCM and siHIF-2α from MCS

The in vitro release profiles of CCM and siHIF-2α from MCS NPs were recorded to evaluate the ability of MIL-101-NH_2_ as scaffolds for sustained delivery. According to the standard curve line (Additional file 1: Fig. S5) of CCM in PBS + 1% v/v Tween 80) at different pH values (5.0, 6.5, and 7.4). As shown in Fig. 1H, MC NPs presented a retarded drug release profile, with merely 21.9% ± 1.4% of CCM, even after 300 h in PBS-Tween 80 (pH = 7.4). However, this result was significantly improved in acidic pH, and the cumulative drug release reached 59.7% ± 1.8% at the same time. In addition, the inset of Fig. 1H clearly showed the differences in color of three samples with varying pH values. This pH-induced release property of MIL-101-NH_2_ could reduce premature drug release in normal chondrocytes and promote drug release in the acidic microenvironments of the inflamed chondrocytes, which is highly beneficial for chronic inflammation treatment.

Likewise, as shown in Fig. 1I, it was found that all MSC NPs released siCy5 for at least seven days with minimal initial burst release. Additionally, about 69% of the siCy5 were diffused from pH 5.0, 6.5, and 7.4 at Day 7. The highest cumulative release rate was found in the lowest pH at Day 7. Meanwhile, this pH-stimulated and sustained release makes it an ideal candidate for OA treatment.

### The stability of MCS NPs

Another important consideration was the stability of MCS NPs against salty ions in the synovium. To evaluate the stability of the MCS NPs in vitro, we measured their particle sizes during incubation in PBS, DMEM, and FBS for a predetermined duration (Additional file 1: Fig. S6A and B). The particle sizes of MCS NPs exhibited nearly unchanged particle size during the 14 days of incubation in these solutions, indicating that MCS NPs exhibited excellent stability against salty ions. Then, the PXRD patterns (Additional file 1: Fig. S7) further confirmed that the structures of MCS NPs exhibited no obvious changes during 14 days in various solutions. These results indicated that MCS NPs could remain structurally stable, even in the complex OA microenvironment.

### In vitro cytotoxicity of MIL-101-NH_2_@CCM-siRNA complex

**Fig. 2:**
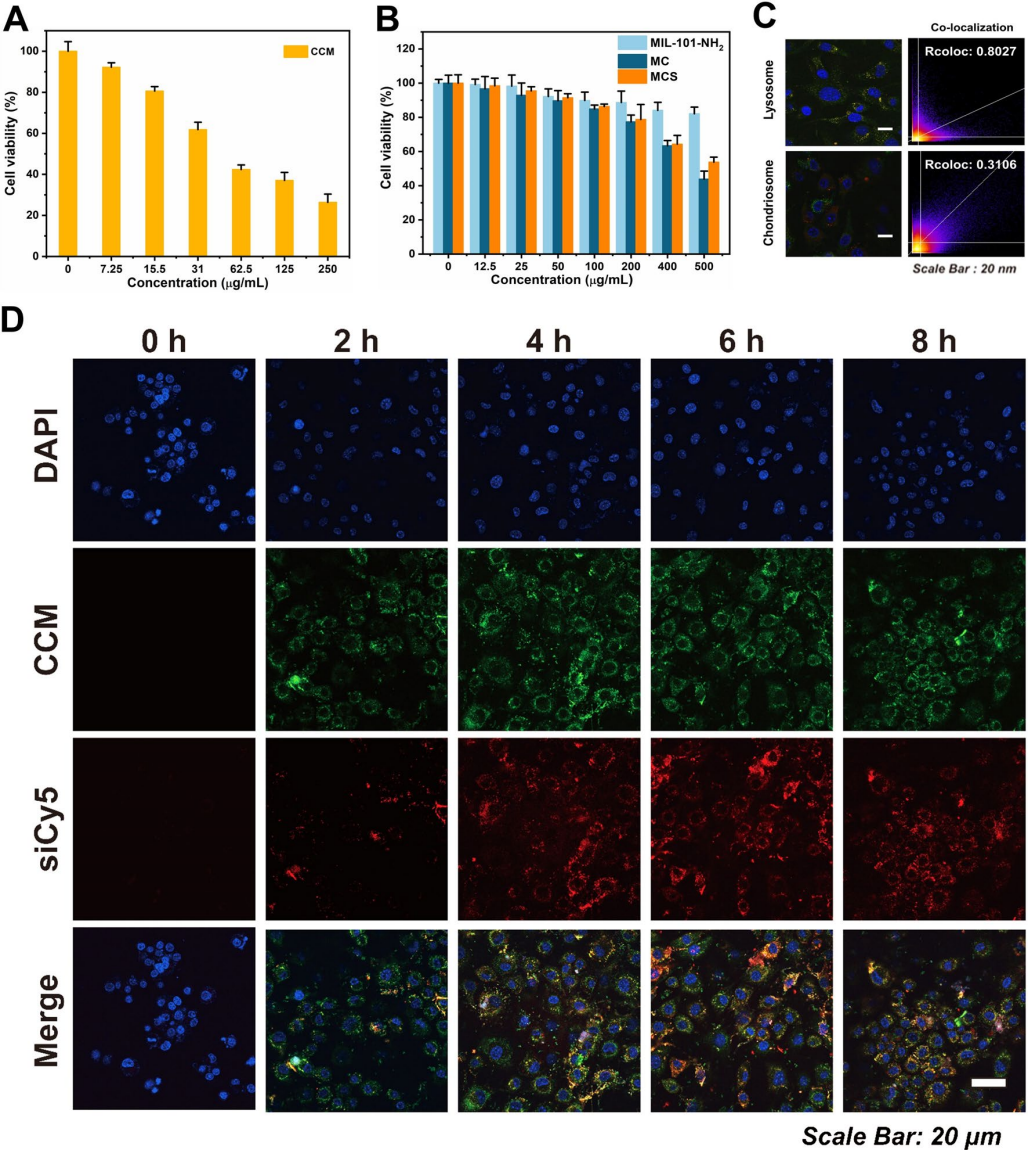
**A** Cell viability of the chondrocytes treated with CCM for 24 h. **B** Cell viabilities of the chondrocytes treated with MIL-101-NH_2_, MC and MCS NPs for 24 h. **C** The lysosome and chondriosome co-localization of MCS in chondrocytes. **D** CLSM images showing the internalization of MIL-101-NH_2_@CCM-siCy5 to the cytoplasm of chondrocytes

The cell viabilities of the components of MCS NPs were evaluated by MTT tests on chondrocytes before further biomedical applications. According to Fig. 2A, CCM had significant cytotoxicity at relatively low concentration (10 μg/mL) against the chondrocytes. However, MIL-101-NH_2_ (Fig. 2B) had no obvious cytotoxicity against chondrocytes even at high concentrations (400 μg/mL). The CCM encapsulation in MIL-101-NH_2_, significantly improved the biocompatibility of CCM (100 μg/mL). At the same time, the MCS NPs also exhibited good biocompatibility, and cell viabilities were all greater than 80% at certain concentrations. These results are predictable since the in vitro analysis was operated after 48 h. However, MIL-101-NH_2_ requires 300 h to release its total drug load. Additionally, as per the release profiles shown in Fig. 1H at 48 h, only 29.7%, 19.5%, and 10.3% of all drug load can be released at pH 5.0, pH 6.5 and pH 7.4, respectively.

Besides, during the extended incubation period of 3 days, almost no dead cells were detected on the live/dead staining assay (Additional file 1: Fig. S8), and the inflammatory chondrocytes were simulated by post-incubated with 10 ng/mL IL-1β for 24 h. These results suggest that MCS NPs have excellent compatibility with chondrocytes.

### Cellular uptake and lysosomal escape of MCS NPs

**Fig. 3:**
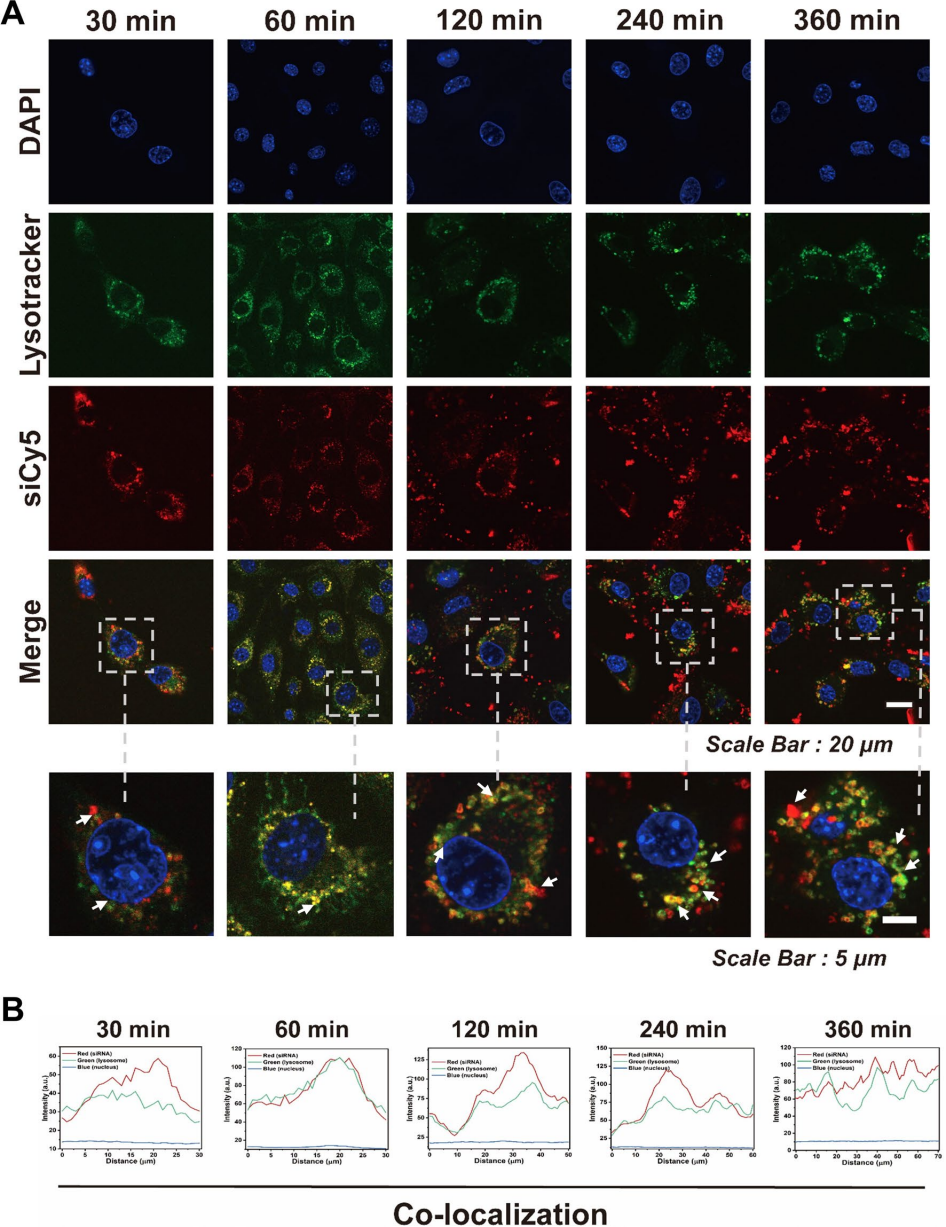
**A** Time-dependent lysosome escape of siCy5 in chondrocytes. **B** Time-dependent change of the co-localization rate between siCy5 and released CCM

For further investigating the pathway of MCS NPs delivery into the chondrocytes, the co-localization of siCy5 with lysosome or mitochondria was evaluated by the CLSM. As shown in Fig. 2C, compared with the mitochondria group, the co-localization rate between MCS NPs and lysosomes was more significant. This indicated that the cellular uptake of nanomaterials is mainly through the lysosomal pathway. Then, due to the intrinsic green fluorescence of CCM, the red fluorescence of siCy5, and the blue fluorescence of DAPI were utilized for nucleic acid (nucleus) staining. The cellular uptake of MSC NPs was observed by CLSM. In order to initiate internalization and transfection processes, MCS NPs must first cross cell membranes. For effective siRNA-mediated gene silencing, high levels of siRNA uptake and endosomal escape were essential [60]. As shown in Fig. 2D, the fluorescence intensity in chondrocytes reached saturation after incubation with MCS NPs for 6 h, indicating that MCS NPs can be effectively uptaken by the inflammatory chondrocytes. Additionally, it was crucial to escape from the lysosome after the successful internalization of siRNA for intracellular siRNA delivery to be effective. As shown in Fig. 3A, there were amounts of siCy5 (red) that apparently overlapped with the lysosome (green) in chondrocytes after 60 min of incubation, indicating that MIL-101-NH_2_@CCM-siCy5 were internalized inside the lysosome of chondrocytes. After 2 h of incubation, the majority of the siCy5 (red) and lysosome tracker (green) fluorescence in the cytoplasm were separated, indicating that the siCy5 escaped from the entrapment of lysosome and then accumulated in the cytoplasm. As a consequence of these results, we speculated that vacant Fe(III) ions of MIL-101-NH_2_ have a strong affinity for the phosphate ions of siCy5. Therefore, when the high concentration of phosphate ion in lysosomes triggered the internalized MIL-101-NH_2_ collapse, the lysosome structure turned to be unstable because of the high binding between the released Fe^3+^ and the phosphate group on the lysosome membrane, so that the siRNA could reduce enzyme degermation and successfully escape. Additionally, relative fluorescence intensity analysis (Fig. 3A), in the areas signed by the white arrow in Fig. 3A, was performed accordingly. Finally, based on the CLSM images, the time-dependent lysosome/siCy5 colocalization studies were investigated (Fig. 3B).

### ROS production induced by MIL-101-NH_2_

An assessment of ROS generated by MIL-101-NH_2_ was conducted using DCFH fluorescence. Incubation of chondrocytes with Rosup, the cytoplasm of chondrocytes displayed intensive green fluorescence, indicating that the level of ROS in the cells increased. For comparison, Additional file 1: Fig. S9 showed that the MIL-101-NH_2_ group exhibited only extremely weak green fluorescence by DCFH, similar to the control group, indicating that the amount of ROS induced by MIL-101-NH_2_ was negligible after incubation for 24 h. Consequently, MIL-101-NH_2_ did not appear to have any passive effect on chondrocytes, indicating that it was compatible for utilization in OA microenvironments.

### The therapeutic effects on IL-1β-induced inflamed chondrocytes

**Fig. 4:**
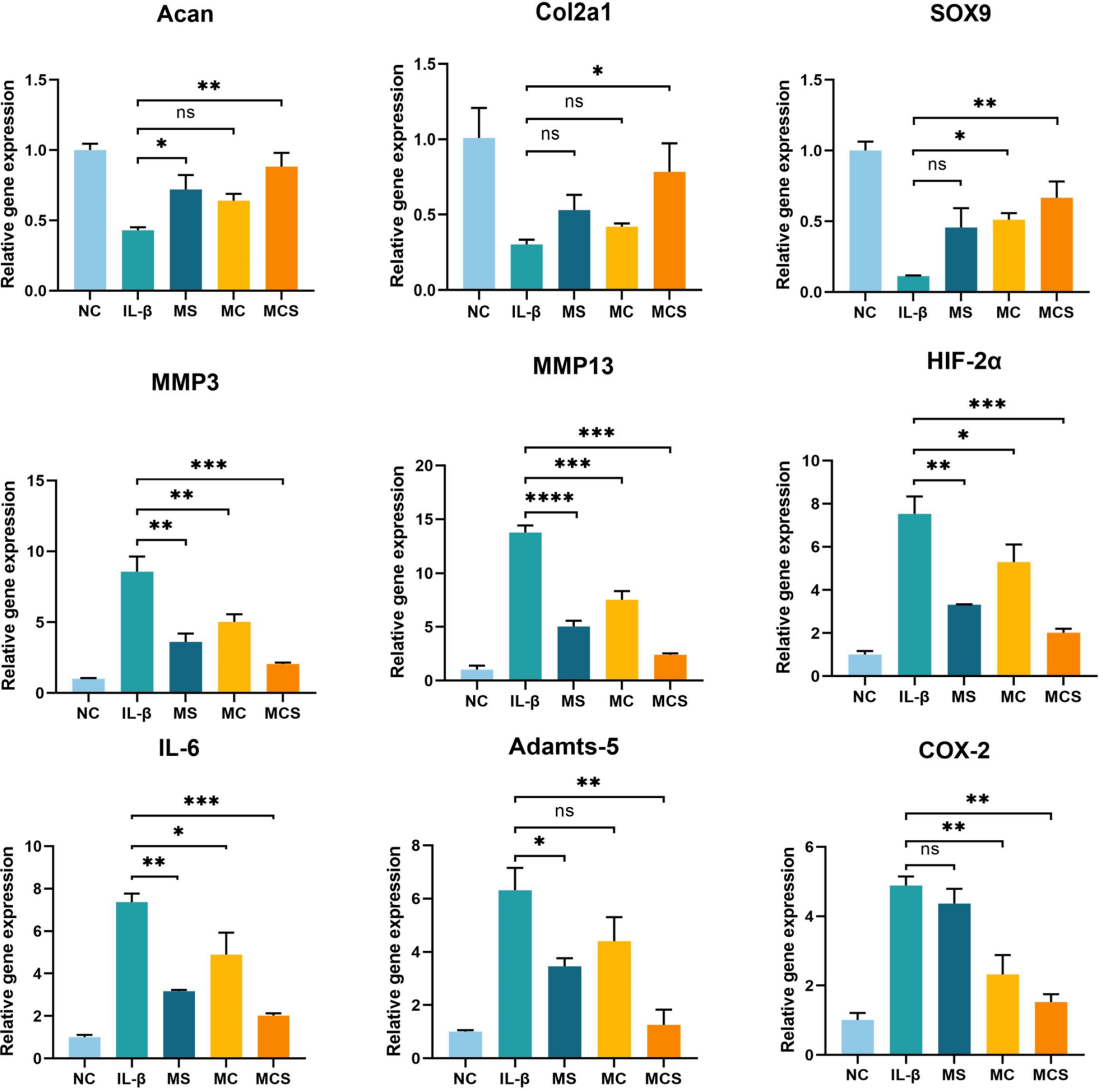
Relative mRNA levels of chondrogenic markers (Acan, Col2a1, and SOX9), OA-relative genes (MMP3, MMP13, HIF-2α, IL-6, Adamts-5, and COX-2). Data are presented herein as the mean ± SD (n = 3). *p < 0.05; **p < 0.01; ***p < 0.001

To investigate the therapeutic effects of MCS NPs, the expression of cartilage-specific markers (including Acan, Col2a1, and SOX9) and OA-related catabolic markers (including Adamts-5, COX-2, IL-6, MMP3, MMP13, and HIF-2α) were further evidenced by using the qRT–PCR assay (Fig. 4) first. The expression of the cartilage-specific markers in IL-1β group was the lowest in all groups, with an obvious decrease of 43.1% for Acan, 30.3% for Col2a1, and 89.3% for SOX9 compared with the NC group. In contrast, the expression of OA-related genes in the IL-1β group was sharply up-regulated compared with those in the other groups (Fig. 4), and slightly down-regulated in treatment groups. Particularly, the results showed that MCS NPs significantly down-regulated the expression of MMP3, MMP13, HIF-2α, IL-6, Adamts-5, and COX-2 by 76.1%, 82.5%, 82.4%, 72.5%, 80.9%, and 68.7% and up-regulated the expression of Acan, Col2a1, and SOX9 by 45.3%, 48.4%, and 56.7%, respectively compared with the IL-1β group after treatment for 24 h (Fig. 4). These data ultimately supported that MCS remarkably down-regulated the level of inflammatory cytokines and alleviated cartilage degeneration compared with other treatment groups.

**Fig. 5:**
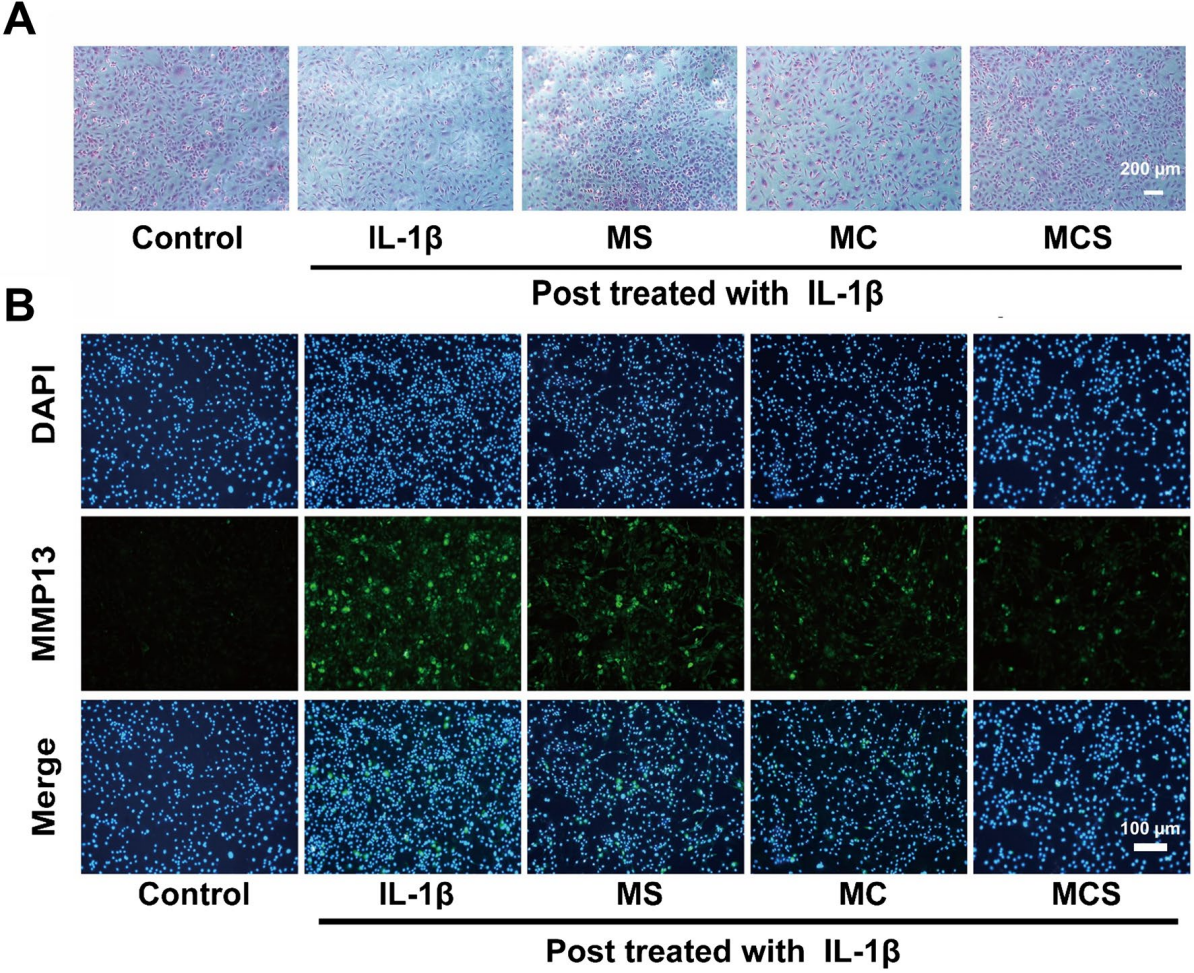
**A** Safranin O staining of chondrocytes in vitro. **B** The expression of MMP13 was observed by immunofluorescence staining

According to the safranin O staining assay (Fig. 5A), the IL-1β group induced weaker positivity (red) than the control group, which indicated that glycosaminoglycan (GAG) production rapidly decreased in OA models. However, the MCS group demonstrated the highest abundance and homogeneity of GAG after 24 h of incubation, which further confirmed the above qRT–PCR results. Additionally, the expression of OA-related biomarkers was also investigated by immunofluorescence staining to further confirm the OA therapeutic effect of MCS NPs.

The results showed strong positive staining of MMP13 in the IL-1β, MC, MS, and MCS groups, particularly in the IL-1β group (Fig. 5B). The MMP13, which plays a crucial role in the development of OA, was less positively secreted in the MCS group than in the other treatment groups, indicating that MCS NPs could synergistically and effectively suppress the inflammatory impairment induced by IL-1β in vitro. In other words, MCS NPs could inhibit the degradation of cartilage matrix, thereby protecting chondrocytes, amplifying the anti-inflammatory effect of the pathological microenvironment associated with OA.

### MIL-101-NH_2_@CCM-siRNA promotes cartilage regeneration in vivo

**Fig. 6:**
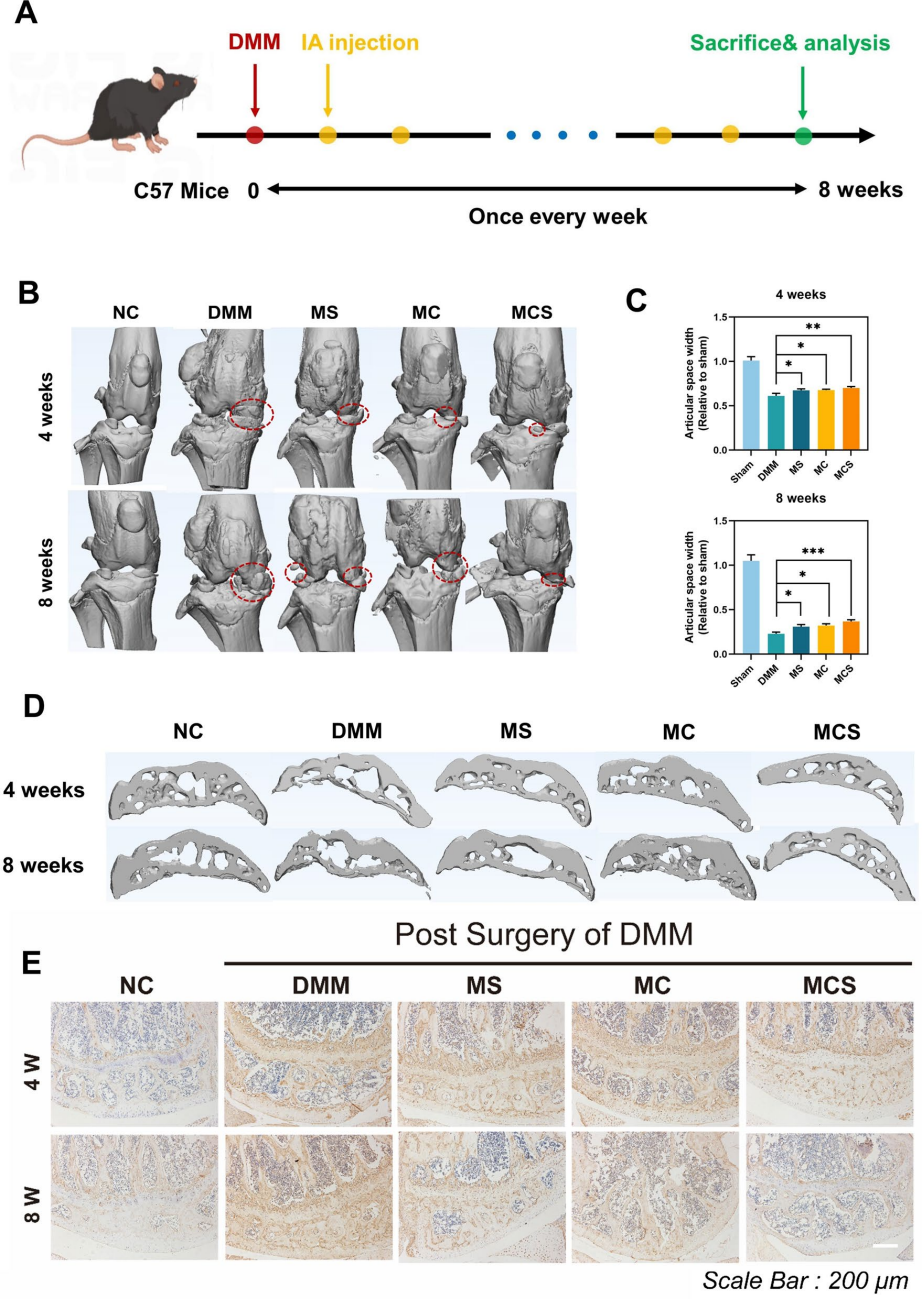
**A** Schedule of the establishment and treatment of OA mice. **B** The reconstructed micro-CT images revealed osteophyte formation to evaluate the therapeutic effect of MC NPs, MS NPs and MCS NPs on OA mice after treatment for four and eight weeks. **C** The relative articular space width of the medial compartments of mouse knee joints at four and eight weeks after surgery. n = 4. *p < 0.05. **D** Representative micro-CT reconstruction sagittal images of subchondral bone at weeks 4 and 8 after treatment. **E** Immunohistochemical staining of MMP13 in cartilage after treatment at weeks 4 and 8

The DMM surgery has been widely operated to establish mice models of OA. The treatment schedule is shown in Fig. 6A. One week after DMM surgery, the mice were intra-articular injected with saline, MC, MS and MCS (all premixed with HA) once a week. Finally, the mice were sacrificed after treatment for four and eight weeks. Before that, the body weight of mice was measured for four and eight weeks (Additional file 1: Fig. S10).

As shown in Fig. 6B, extensive pathological changes were observed in the DMM group, including joint space narrowing, cartilage destruction, subchondral bone sclerosis, and osteophyte formation. According to the CT images, the widths of the articular space and the volumes of osteophytes were both analyzed. The reconstructed micro-CT images directly showed that osteophyte formation at weeks 4 and 8, as indicated with red circles, was significantly reduced in the MCS group, compared with DMM, MC, and MS groups (Fig. 6B). All groups displayed an increase in the osteophyte volume, but the MCS group had a lower value in comparison with the other groups, indicating that it had an improved therapeutic effect in reducing DMM-induced osteophyte formation. After the treatment for four and eight weeks, the articular space widths of the mice’s knee joints were also estimated, as shown in Fig. 6C. After four weeks of treatment, the articular space widths of the MS, MC, and MCS groups were similar and slightly higher than the DMM group. The joint space significantly narrowed after eight weeks in the five groups, however, the MCS group had a wider articular space than the DMM, MC group, and MS groups. These results further indicated that the synergistic therapy (MCS NPs in the HA solution), which simultaneously improved intra-articular lubrication and inhibits the up-regulation of pro-inflammatory cytokines, efficiently obstructed the development of OA. The CT section imaging results of the subchondral bone of mice were also shown in Fig. 6D. Subchondral bone trabeculae in the DMM group became wide and symphyseal, some were arranged unevenly, and the mesh structure disappeared. The trabecular bone became narrowed, and the fusion phenomenon was improved, in the MC, MS, and MCS groups, especially in the MCS group. These results indicated that the structure of bone and joint has improved more effectively in the MCS group, compared with the other groups.

Additionally, the expression levels of MMP13, the typical marker of articular cartilage, were evaluated via immunohistochemical assays (Fig. 6E). The expression of MMP13 was increased in the DMM group, and this change was reversed in the MC, MS and MCS groups. Remarkably, the expression of MMP13 was the lowest in the MCS group compared with the other treatment groups. These results also showed that the combination of CCM with siHIF-2α produced synergistic action in the treatment of OA.

**Fig. 7:**
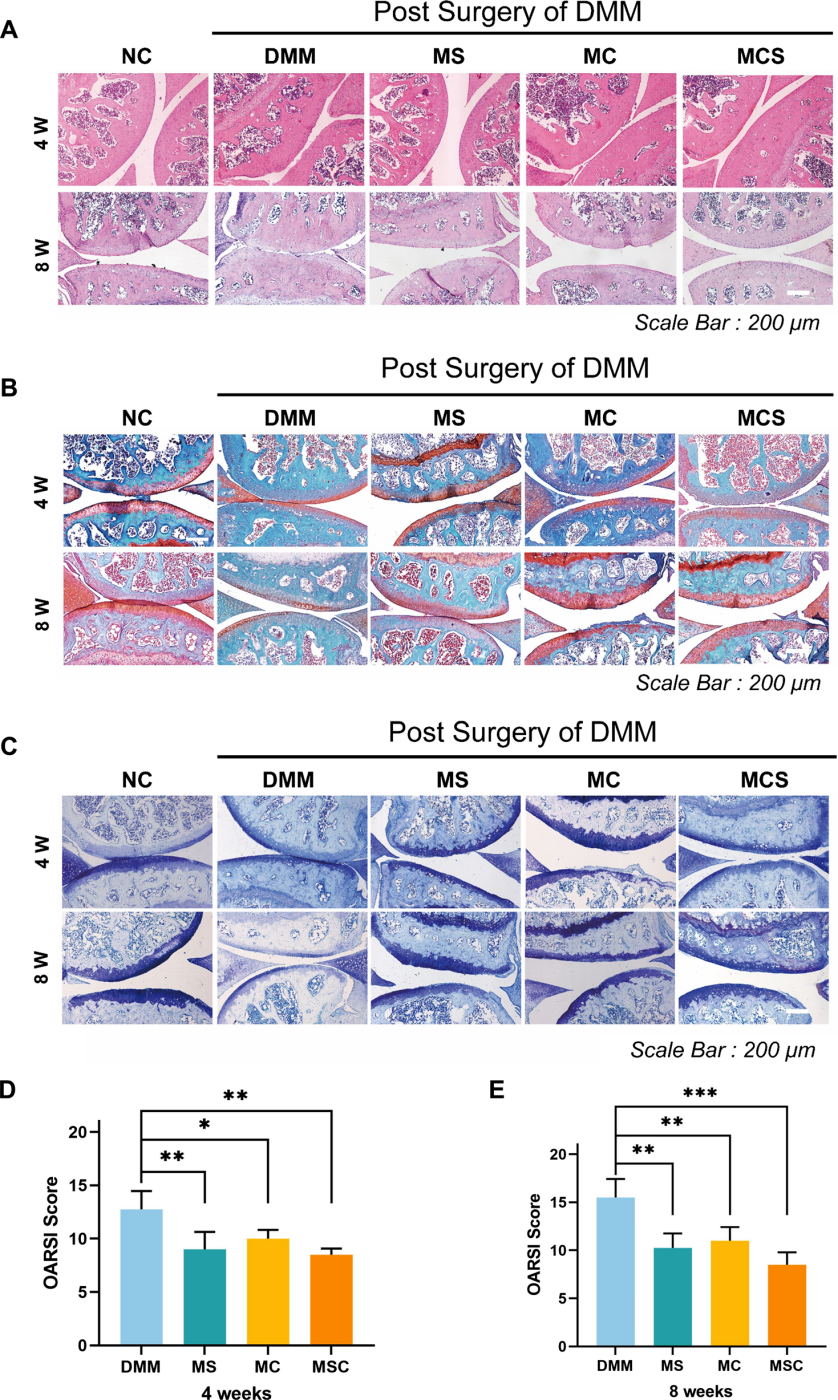
Representative **A** H&E staining, **B** safranin O/fast green staining, and **C** toluidine blue staining of the cartilage sections after treatment of osteoarthritis using saline, MC, MS, and MCS NPs at weeks 4 and 8 after surgery. Score based on OARSI after treatment for **D** four weeks and **E** eight weeks

To further investigate the therapeutic effect of MCS on OA, we performed histological assessments of cartilage tissues by H&E staining, safranin O/fast green staining, and toluidine blue staining. As shown in Fig. 7A, typical OA features, such as vertical fissure, erosion denudation, surface discontinuity and deformation, were observed in the DMM group. Compared to the DMM group, all treatment groups showed varying degrees of improvement in matrix staining, morphological changes, and integrity of tidemarks. Specifically, the MCS group exhibited a greater degree of morphological integrity with fewer severe lesions, less surface denudation, and an increase in tissue cellularity. Additionally, after DMM surgery for four and eight weeks, the MCS group presented more intense safranin O/fast green and toluidine blue positive staining compared with other treatment groups. Based on the results of safranin O/fast green staining and toluidine blue staining, GAG in the five groups was also analyzed (Fig. 7B and C). The MCS group had the best outcome with regard to the GAG level, indicating that MCS can effectively protect articular cartilage during the development of OA in terms of attenuation of cartilage matrix depletion and GAG deposition.

Furthermore, the result of OARSI scores revealed a significant reduction in value for the MCS group compared with the other groups during treatment. After four weeks treatment (Fig. 7D), the MS, MC, and MCS groups respectively showed 29.4%, 21.6%, and 33.3% reduction related to the DMM group. However, after 8 weeks of treatment (Fig. 7E), the MS and MC groups had lower OARSI scores than the DMM group and showed better results with approximately 33.8% and 29.1% reductions, and the MCS group had the highest OARSI score reduction related to DMM group (45.2%) which indicated that CCM and siHIF-2α had a synergistic effect on OA treatment.

## Conclusions

In summary, we have successfully constructed MCS NPs for the co-delivery of CCM and siHIF-2α to establish a pH-responsive system for OA therapy. This multi-modal therapeutic system could improve the long-term stability and bioavailability of CCM and siHIF-2α in the joint cavity. Specifically, the MCS NPs could promote siHIF-2α escape from the lysosome to silence HIF-2α genes in chondrocytes. Owing to the acidic microenvironment in impaired joints, the CCM and siHIF-2α released from scaffolds could alleviate the progression of OA by synergistically downregulating the expression of OA-related catabolic markers and upregulating the expression of cartilage-specific markers in chondrocytes. Altogether, the MCS NPs exhibit remarkable effects on anti-inflammation and cartilage regeneration by combining drug therapy and gene therapy. Because the pathogenesis of OA is complicated, diverse actually, and sometimes related to microenvironmental factors, this multi-modal therapeutic system may provide a promising strategy for OA treatment.

## Supplementary Information

**Additional file 1: Figure S1. **SEM and TEM of MIL-101-NH_2_ NPs. **Figure S2. **The UV-Vis absorbance spectrum and standard curve of CCM. **Figure S3. **The fluorescence spectrum of MC NPs with different DLC(CCM) in siCy5 solution. **Figure S4. **Gel retardation assay of MCS with different DLCs of CCM. **Figure S5.** The standard curve line of CCM in PBS-Tween 80 with different pH values. **Figure S6.** The hydrodynamic diameters of MCS NPs incubated in PBS, DMEM/F12, and FBS for 14 days. **Figure S7.** The PXRD patterns of MCS NPs incubated in PBS, DMEM/F12, and FBS for 14 days. **Figure S8. **Live/dead assay of MCS NPs in chondrocytes. **Figure S9. **ROS generation of intracellular induced by MIL-101-NH_2_. **Figure S10. **Relative body weight changes of mice after DMM surgery. **Table S1. **The DLCs and DLEs of different weight ratios of MIL-101-NH_2_ and CCM. **Table S2.** Different siRNA and objectives used in the experiments. **Table S3. **DLS and zeta potential of MIL-101-NH_2_@CCM-siRNA_X_ complexes at different weight ratios. **Equation S1-2.** Formulas to calculate loaded CCM (DLC and DLE) in MIL-101NH_2_. **Equation S3. **Formula to calculate percent of CCM and siRNA release.

## Data Availability

The datasets used and/or analyzed during current study are available from corresponding author on reasonable request.

## References

[1] Sanchez-Lopez E, Coras R, Torres A, Lane NE, Guma M (2022). Synovial inflammation in osteoarthritis progression. Nat Rev Rheumatol.

[2] Chen Y, Zhao J, Lao S, Lu WW (2019). Association between bisphosphonate use and risk of undergoing knee replacement in patients with osteoarthritis. Ann Rheum Dis.

[3] Sandell LJ (2012). Etiology of osteoarthritis: genetics and synovial joint development. Nat Rev Rheumatol.

[4] Martel-Pelletier J, Barr AJ, Cicuttini FM, Conaghan PG, Cooper C, Goldring MB, Goldring SR, Jones G, Teichtahl AJ, Pelletier JP (2016). Osteoarthritis. Nat Rev Dis Primers.

[5] Zhang M, Egan B, Wang J (2015). Epigenetic mechanisms underlying the aberrant catabolic and anabolic activities of osteoarthritic chondrocytes. Int J Biochem Cell Biol.

[6] Yu P, Li Y, Sun H, Ke X, Xing J, Zhao Y, Xu X, Qin M, Xie J, Li J. Cartilage-inspired hydrogel with mechanical adaptability, controllable lubrication, and inflammation regulation abilities. ACS Appl Mater Interfaces. 2022;14(23): 27360-27370. 10.1021/acsami.2c04609 35658410

[7] Faust HJ, Sommerfeld SD, Rathod S, Rittenbach A, Ray Banerjee S, Tsui BMW, Pomper M, Amzel ML, Singh A, Elisseeff JH (2018). A hyaluronic acid binding peptide-polymer system for treating osteoarthritis. Biomaterials..

[8] Bottini M, Bhattacharya K, Fadeel B, Magrini A, Bottini N, Rosato N (2016). Nanodrugs to target articular cartilage: an emerging platform for osteoarthritis therapy. Nanomedicine.

[9] Altman R, Asch E, Bloch D, Bole G, Borenstein D, Brandt K, Christy W, Cooke TD, Greenwald R, Hochberg M (1986). Development of criteria for the classification and reporting of osteoarthritis. Classification of osteoarthritis of the knee. Diagnostic and Therapeutic Criteria Committee of the American Rheumatism Association. Arthritis Rheum.

[10] Fraenkel L, Buta E, Suter L, Dubreuil M, Levy C, Najem C, Brennan M, Corn B, Kerns R, Goulet J (2020). Nonsteroidal anti-inflammatory drugs vs cognitive behavioral therapy for arthritis pain. JAMA Intern Med.

[11] Cao P, Li Y, Tang Y, Ding C, Hunter DJ (2020). Pharmacotherapy for knee osteoarthritis: current and emerging therapies. Expert Opin Pharmacother.

[12] da Costa BR, Reichenbach S, Keller N, Nartey L, Wandel S, Jüni P, Trelle S (2017). Effectiveness of non-steroidal anti-inflammatory drugs for the treatment of pain in knee and hip osteoarthritis: a network meta-analysis. Lancet.

[13] Kang C, Jung E, Hyeon H, Seon S, Lee D (2020). Acid-activatable polymeric curcumin nanoparticles as therapeutic agents for osteoarthritis. Nanomedicine.

[14] Wang Z, Jones G, Winzenberg T, Cai G, Laslett LL, Aitken D, Hopper I, Singh A, Jones R, Fripp J, Ding C, Antony B (2020). Effectiveness of *Curcuma*
*longa* extract for the treatment of symptoms and effusion-synovitis of knee osteoarthritis : a randomized trial. Ann Intern Med.

[15] Zhang M, Zhang X, Tian T, Zhang Q, Wen Y, Zhu J, Xiao D, Cui W, Lin Y (2022). Anti-inflammatory activity of curcumin-loaded tetrahedral framework nucleic acids on acute gouty arthritis. Bioact Mater.

[16] Sun HJ, Ren XS, Xiong XQ, Chen YZ, Zhao MX, Wang JJ, Zhou YB, Han Y, Chen Q, Li YH, Kang YM, Zhu GQ (2017). NLRP3 inflammasome activation contributes to VSMC phenotypic transformation and proliferation in hypertension. Cell Death Dis.

[17] Anand P, Kunnumakkara AB, Newman RA, Aggarwal BB (2007). Bioavailability of curcumin: problems and promises. Mol Pharm.

[18] Wu Y, Li J, Zeng Y, Pu W, Mu X, Sun K, Peng Y, Shen B (2022). Exosomes rewire the cartilage microenvironment in osteoarthritis: from intercellular communication to therapeutic strategies. Int J Oral Sci.

[19] Pena SA, Iyengar R, Eshraghi RS, Bencie N, Mittal J, Aljohani A, Mittal R, Eshraghi AA (2020). Gene therapy for neurological disorders: challenges and recent advancements. J Drug Target.

[20] Rai MF, Pan H, Yan H, Sandell LJ, Pham CTN, Wickline SA (2019). Applications of RNA interference in the treatment of arthritis. Transl Res.

[21] Feng N, Guo F (2020). Nanoparticle-siRNA: a potential strategy for rheumatoid arthritis therapy?. J Control Release.

[22] Yang S, Kim J, Ryu JH, Oh H, Chun CH, Kim BJ, Min BH, Chun JS (2010). Hypoxia-inducible factor-2alpha is a catabolic regulator of osteoarthritic cartilage destruction. Nat Med.

[23] Zhang FJ, Luo W, Lei GH (2015). Role of HIF-1alpha and HIF-2alpha in osteoarthritis. Joint Bone Spine.

[24] Pi Y, Zhang X, Shao Z, Zhao F, Hu X, Ao Y (2015). Intra-articular delivery of anti-Hif-2alpha siRNA by chondrocyte-homing nanoparticles to prevent cartilage degeneration in arthritic mice. Gene Ther.

[25] Pasupneti S, Tian W, Tu AB, Dahms P, Granucci E, Gandjeva A, Xiang M, Butcher EC, Semenza GL, Tuder RM, Jiang X, Nicolls MR (2020). Endothelial HIF-2alpha as a key endogenous mediator preventing emphysema. Am J Respir Crit Care Med.

[26] Yang Q, Zhou Y, Cai P, Fu W, Wang J, Wei Q, Li X (2019). Up-regulated HIF-2alpha contributes to the osteoarthritis development through mediating the primary cilia loss. Int Immunopharmacol.

[27] Pecot CV, Calin GA, Coleman RL, Lopez-Berestein G, Sood AK (2011). RNA interference in the clinic: challenges and future directions. Nat Rev Cancer.

[28] Whitehead KA, Langer R, Anderson DG (2009). Knocking down barriers: advances in siRNA delivery. Nat Rev Drug Discov.

[29] Feng J, Yu W, Xu Z, Hu J, Liu J, Wang F (2020). Multifunctional siRNA-laden hybrid nanoplatform for noninvasive PA/IR dual-modal imaging-guided enhanced photogenetherapy. ACS Appl Mater Interfaces.

[30] Teplensky MH, Fantham M, Poudel C, Hockings C, Lu M, Guna A, Aragones-Anglada M, Moghadam PZ, Li P, Farha OK, de Quirós Fernández SB (2019). A highly porous metal-organic framework system to deliver payloads for gene knockdown. Chem.

[31] Zhang W, Liang L, Yuan X, Wang F, Shan X, Li J, Wang Z, Yang X (2021). Intelligent dual responsive modified ZIF-8 nanoparticles for diagnosis and treatment of osteoarthritis. Mater Des.

[32] Tao W, Wang J, Parak WJ, Farokhzad OC, Shi J (2019). Nanobuffering of pH-responsive polymers: a known but sometimes overlooked phenomenon and its biological applications. ACS Nano.

[33] Li C, Li H, Wang Q, Zhou M, Li M, Gong T, Zhang Z, Sun X (2017). pH-sensitive polymeric micelles for targeted delivery to inflamed joints. J Control Release.

[34] Zhang M, Hu W, Cai C, Wu Y, Li J, Dong S (2022). Advanced application of stimuli-responsive drug delivery system for inflammatory arthritis treatment. Mater Today Bio.

[35] Xue S, Zhou X, Sang W, Wang C, Lu H, Xu Y, Zhong Y, Zhu L, He C, Ma J (2021). Cartilage-targeting peptide-modified dual-drug delivery nanoplatform with NIR laser response for osteoarthritis therapy. Bioact Mater.

[36] Du T, Qin Z, Zheng Y, Jiang H, Weizmann Y, Wang X (2019). The “Framework Exchange”-strategy-based MOF platform for biodegradable multimodal therapy. Chem.

[37] Wang L, Zheng M, Xie Z (2018). Nanoscale metal-organic frameworks for drug delivery: a conventional platform with new promise. J Mater Chem B.

[38] Wang S, McGuirk CM, d'Aquino A, Mason JA, Mirkin CA (2018). Metal-organic framework nanoparticles. Adv Mater.

[39] Chen Q, Xu M, Zheng W, Xu T, Deng H, Liu J (2017). Se/Ru-decorated porous metal-organic framework nanoparticles for the delivery of pooled siRNAs to reversing multidrug resistance in taxol-resistant breast cancer cells. ACS Appl Mater Interfaces.

[40] Li L, Han S, Zhao S, Li X, Liu B, Liu Y (2020). Chitosan modified metal-organic frameworks as a promising carrier for oral drug delivery. RSC Adv.

[41] Sun Y, Zheng L, Yang Y, Qian X, Fu T, Li X, Yang Z, Yan H, Cui C, Tan W (2020). Metal-organic framework nanocarriers for drug delivery in biomedical applications. Nanomicro Lett.

[42] Hidalgo T, Alonso-Nocelo M, Bouzo BL, Reimondez-Troitino S, Abuin-Redondo C, de la Fuente M, Horcajada P (2020). Biocompatible iron(iii) carboxylate metal-organic frameworks as promising RNA nanocarriers. Nanoscale.

[43] Karimi Alavijeh R, Akhbari K (2020). Biocompatible MIL-101(Fe) as a smart carrier with high loading potential and sustained release of curcumin. Inorg Chem.

[44] Shen S, Li L, Li S, Bai Y, Liu H (2018). Metal-organic frameworks induce autophagy in mouse embryonic fibroblast cells. Nanoscale.

[45] Rai MF, Pham CT (2018). Intra-articular drug delivery systems for joint diseases. Curr Opin Pharmacol.

[46] Brown S, Kumar S, Sharma B (2019). Intra-articular targeting of nanomaterials for the treatment of osteoarthritis. Acta Biomater.

[47] Maudens P, Seemayer CA, Thauvin C, Gabay C, Jordan O, Allemann E (2018). Nanocrystal-polymer particles: extended delivery carriers for osteoarthritis treatment. Small.

[48] Maudens P, Jordan O, Allemann E (2018). Recent advances in intra-articular drug delivery systems for osteoarthritis therapy. Drug Discov Today.

[49] Xiong F, Qin Z, Chen H, Lan Q, Wang Z, Lan N, Yang Y, Zheng L, Zhao J, Kai D (2020). pH-responsive and hyaluronic acid-functionalized metal-organic frameworks for therapy of osteoarthritis. J Nanobiotechnology.

[50] Ji P, Wang L, Wang S, Zhang Y, Qi X, Tao J, Wu Z (2020). Hyaluronic acid-coated metal-organic frameworks benefit the ROS-mediated apoptosis and amplified anticancer activity of artesunate. J Drug Target.

[51] Sun Q, Bi H, Wang Z, Li C, Wang X, Xu J, Zhu H, Zhao R, He F, Gai S, Yang P (2019). Hyaluronic acid-targeted and pH-responsive drug delivery system based on metal-organic frameworks for efficient antitumor therapy. Biomaterials.

[52] Gupta RC, Lall R, Srivastava A, Sinha A (2019). Hyaluronic acid: molecular mechanisms and therapeutic trajectory. Front Vet Sci.

[53] Kang LJ, Yoon J, Rho JG, Han HS, Lee S, Oh YS, Kim H, Kim E, Kim SJ, Lim YT, Park JH, Song WK, Yang S, Kim W (2021). Self-assembled hyaluronic acid nanoparticles for osteoarthritis treatment. Biomaterials.

[54] Yang Y, Chen Q, Wu JP, Kirk TB, Xu J, Liu Z, Xue W (2018). Reduction-responsive codelivery system based on a metal-organic framework for eliciting potent cellular immune response. ACS Appl Mater Interfaces.

[55] Guo L, Zhong S, Liu P, Guo M, Ding J, Zhou W (2022). Radicals scavenging MOFs enabling targeting delivery of siRNA for rheumatoid arthritis therapy. Small.

[56] Jiang T, Kai D, Liu S, Huang X, Heng S, Zhao J, Chan BQY, Loh XJ, Zhu Y, Mao C, Zheng L (2018). Mechanically cartilage-mimicking poly(PCL-PTHF urethane)/collagen nanofibers induce chondrogenesis by blocking NF-kappa B signaling pathway. Biomaterials.

[57] Dashnyam K, Lee J-H, Singh RK, Yoon J-Y, Lee JH, Jin G-Z, Kim H-W (2021). Optimally dosed nanoceria attenuates osteoarthritic degeneration of joint cartilage and subchondral bone. Chem Eng J.

[58] Glasson SS, Chambers MG, Van Den Berg WB, Little CB (2010). The OARSI histopathology initiative-recommendations for histological assessments of osteoarthritis in the mouse. Osteoarthr Cartil.

[59] Zhang Y, Wang L, Liu L, Lin L, Liu F, Xie Z, Tian H, Chen X (2018). Engineering metal-organic frameworks for photoacoustic imaging-guided chemo-/photothermal combinational tumor therapy. ACS Appl Mater Interfaces.

[60] He C, Lu K, Liu D, Lin W (2014). Nanoscale metal-organic frameworks for the co-delivery of cisplatin and pooled siRNAs to enhance therapeutic efficacy in drug-resistant ovarian cancer cells. J Am Chem Soc.

