# Supplementary material for: A pH-responsive metal-organic framework for the co-delivery of HIF-2α siRNA and curcumin for enhanced therapy of osteoarthritis
Source: J Nanobiotechnology. 2023 Jan 17;21:18. doi: 10.1186/s12951-022-01758-2 (PMC9847079; doi:10.1186/s12951-022-01758-2)
Supplement: Supplementary file 1 — Additional file 1: Figure S1. SEM and TEM of MIL-101-NH2 NPs. Figure S2. The UV-Vis absorbance spectrum and standard curve of CCM. Figure S3. The fluorescence spectrum of MC NPs with different DLC(CCM) in siCy5 solution. Figure S4. Gel retardation assay of MCS with different DLCs of CCM. Figure S5. The standard curve line of CCM in PBS-Tween 80 with different pH values. Figure S6. The hydrodynamic diameters of MCS NPs incubated in PBS, DMEM/F12, and FBS for 14 days. Figure S7. The PXRD patterns of MCS NPs incubated in PBS, DMEM/F12, and FBS for 14 days. Figure S8. Live/dead assay of MCS NPs in chondrocytes. Figure S9. ROS generation of intracellular induced by MIL-101-NH2. Figure S10. Relative body weight changes of mice after DMM surgery. Table S1. The DLCs and DLEs of different weight ratios of MIL-101-NH2 and CCM. Table S2. Different siRNA and objectives used in the experiments. Table S3. DLS and zeta potential of MIL-101-NH2@CCM-siRNAX complexes at different weight ratios. Equation S1-2. Formulas to calculate loaded CCM (DLC and DLE) in MIL-101NH2. Equation S3. Formula to calculate percent of CCM and siRNA release. [file 12951_2022_1758_MOESM1_ESM.doc]

## A pH-Responsive Metal−organic Framework for the Co-delivery of HIF-2α siRNA and Curcumin toward Enhanced Therapy of Osteoarthritis

Zi-Jian Zhang^#a^, Ying-Ke Hou^#a^, Ming-Wa Chen^b^, Xue-Zhao Yu^a^, Si-Yu Chen^a^, Ya-Ru Yue^a^, Xiong-Tian Guo^a^, Jin-Xiang Chen^b^**, Quan Zhou^a^*

^a^ Department of Medical Imaging, Third Affiliated Hospital of Southern Medical University (Academy of Orthopedics Guangdong Province), Southern Medical University, Guangzhou, Guangdong 510630, People’s Republic of China

^b^ NMPA Key Laboratory for Research and Evaluation of Drug Metabolism, Guangdong Provincial Key Laboratory of New Drug Screening, School of Pharmaceutical Sciences, Southern Medical University, Guangzhou 510515, People’s Republic of China

* Corresponding author

** Corresponding author

E-mails: zhouquan3777@smu.edu.cn (Q. Zhou); jxchen@smu.edu.cn (J. X. Chen)

^1^Z.J. Zhang and Y.K. Hou contributed equally to this work.

**Table of Contents**

**Figure S1.** SEM and TEM of MIL-101-NH_2_ NPs

**Figure S2.** The UV-Vis absorbance spectrum and standard curve of CCM

**Figure S3.** The fluorescence spectrum of MC NPs with different DLC(CCM) in siCy5 solution

**Figure S4.** Gel retardation assay of MCS with different DLCs of CCM

**Figure S5.** The standard curve line of CCM in PBS-Tween 80 with different pH values

**Figure S6.** The hydrodynamic diameters of MCS NPs incubated in PBS, DMEM/F12, and FBS for 14 days

**Figure S7.** The PXRD patterns of MCS NPs incubated in PBS, DMEM/F12, and FBS for 14 days

**Figure S8.** Live/dead assay of MCS NPs in chondrocytes

**Figure S9.** ROS generation of intracellular induced by MIL-101-NH_2_

**Figure S10.** Relative body weight changes of mice after DMM surgery

**Table S1.** The DLCs and DLEs of different weight ratios of MIL-101-NH_2_ and CCM

**Table S2.** Different siRNA and objectives used in the experiments

**Table S3.** DLS and zeta potential of MIL-101-NH_2_@CCM-siRNA_X_ complexes at different weight ratios

**Equation S1-2.** Formulas to calculate loaded CCM (DLC and DLE) in MIL-101NH_2_

**Equation S3.** Formula to calculate percent of CCM and siRNA release

**EXPERIMENTAL SECTION**

**
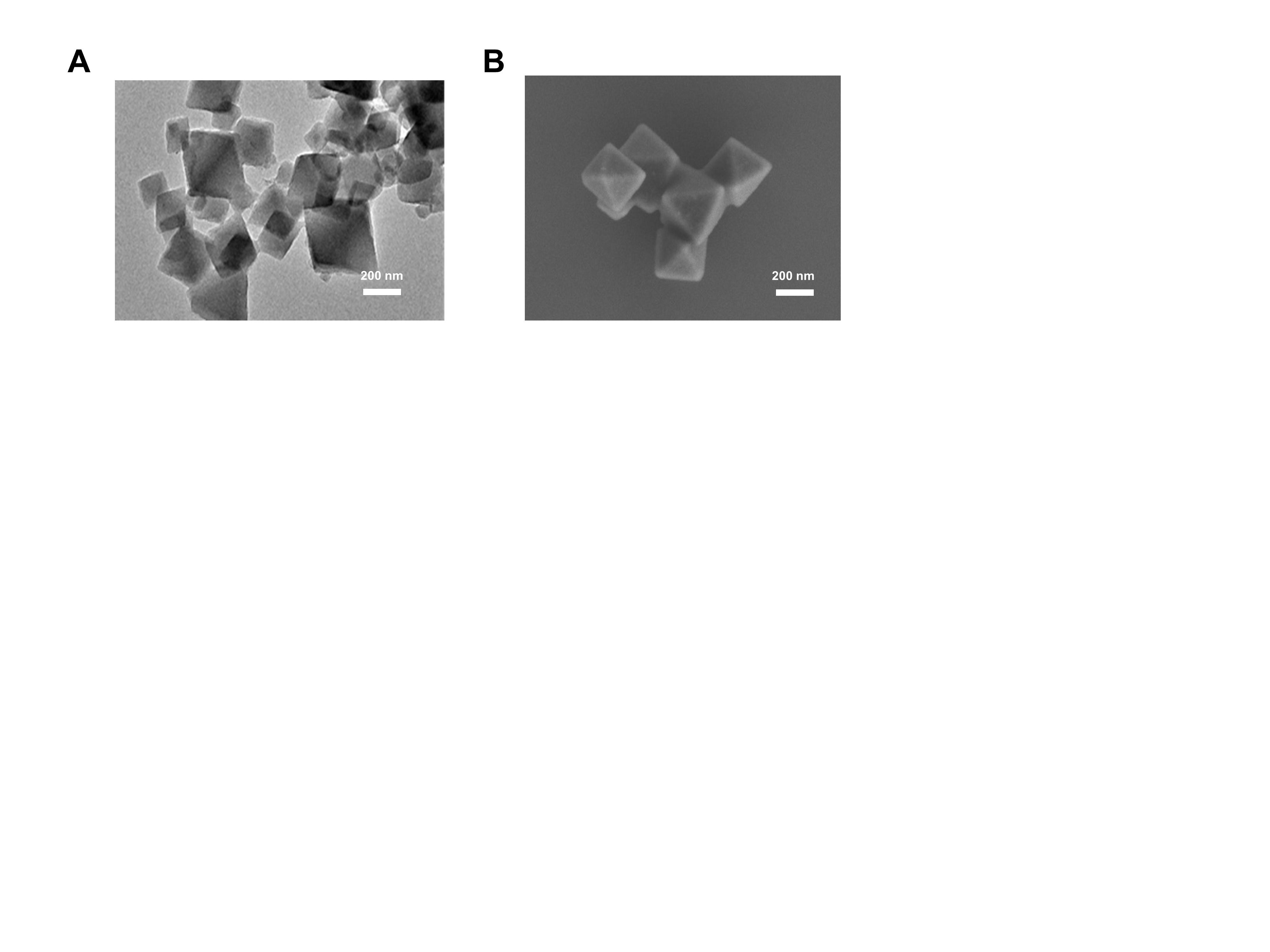
**

**Figure S1 A)** TEM image and **B)** SEM image of MIL-101-NH_2_ NPs. (Scale bar: 200 nm)

**
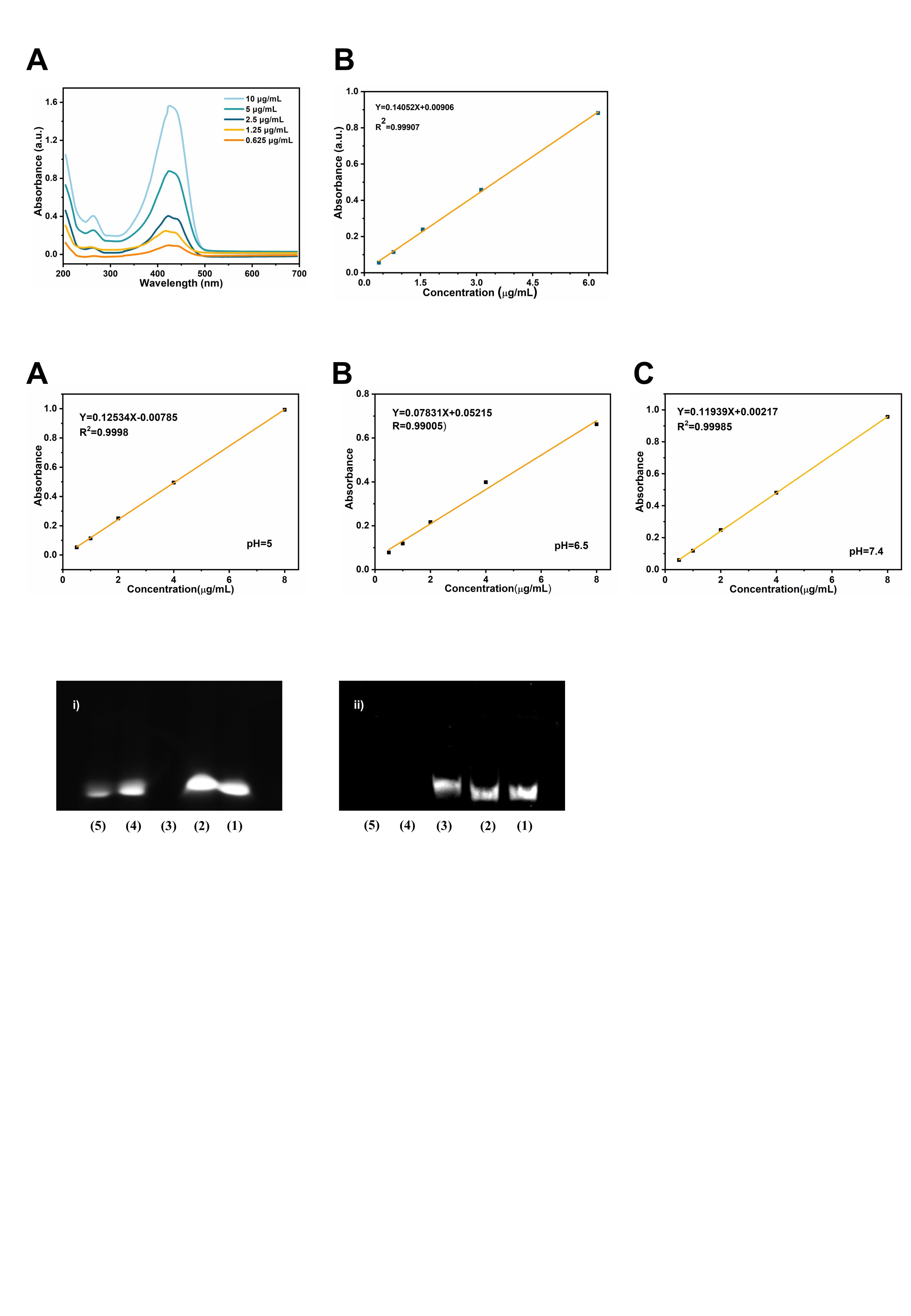
**

**Figure S2. A)** The UV-Vis absorbance spectrum of CCM in ethanol with different concentrations. **B)** The standard curve line of CCM in ethanol.


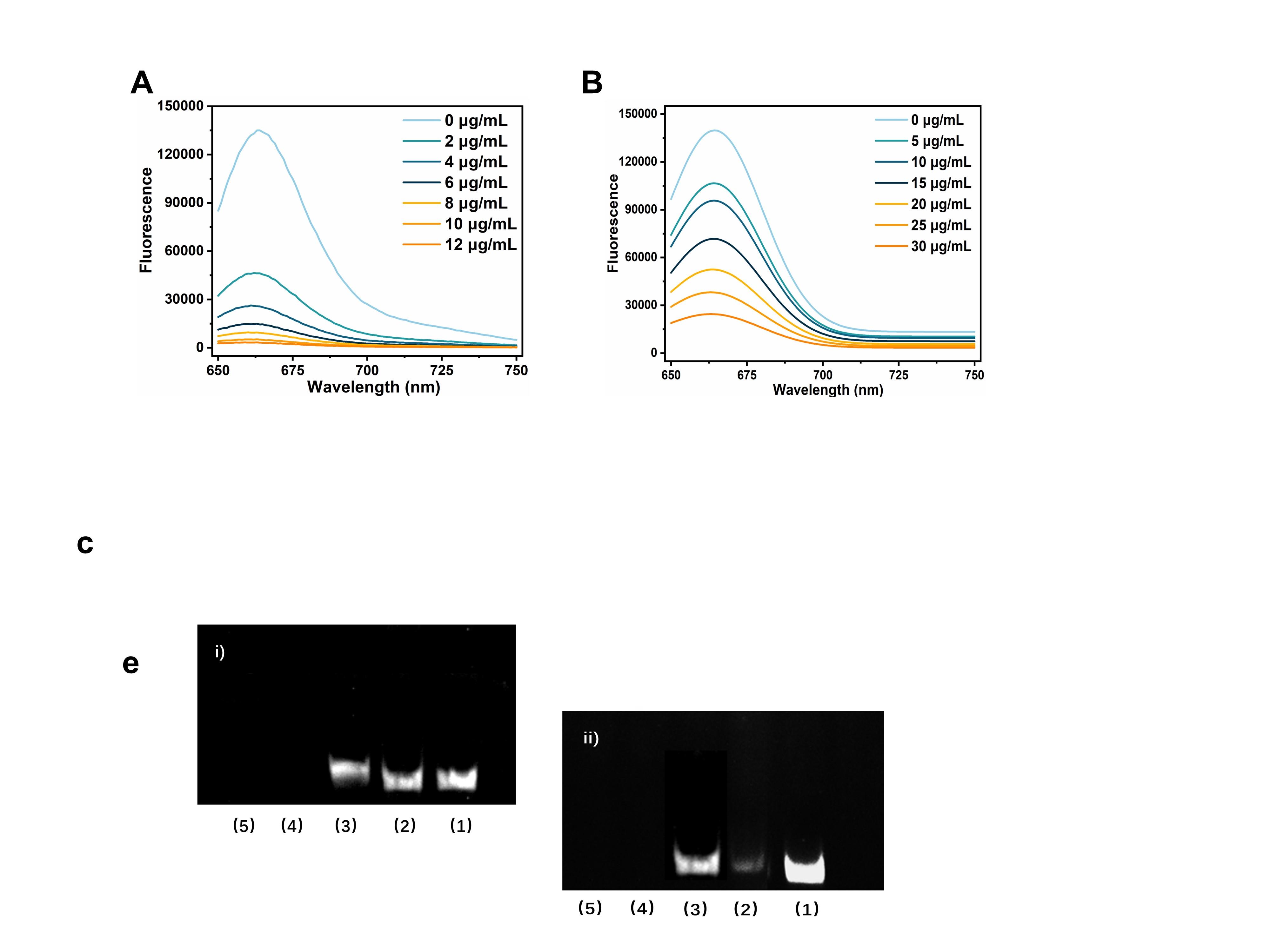


**Figure S3.** The fluorescence spectrum of **A)** MC NPs (DLC of CCM = 25.9%), and **B)** MC NPs (DLC of CCM = 42.5%) adding to the siCy5 solution with 100 nM, respectively.


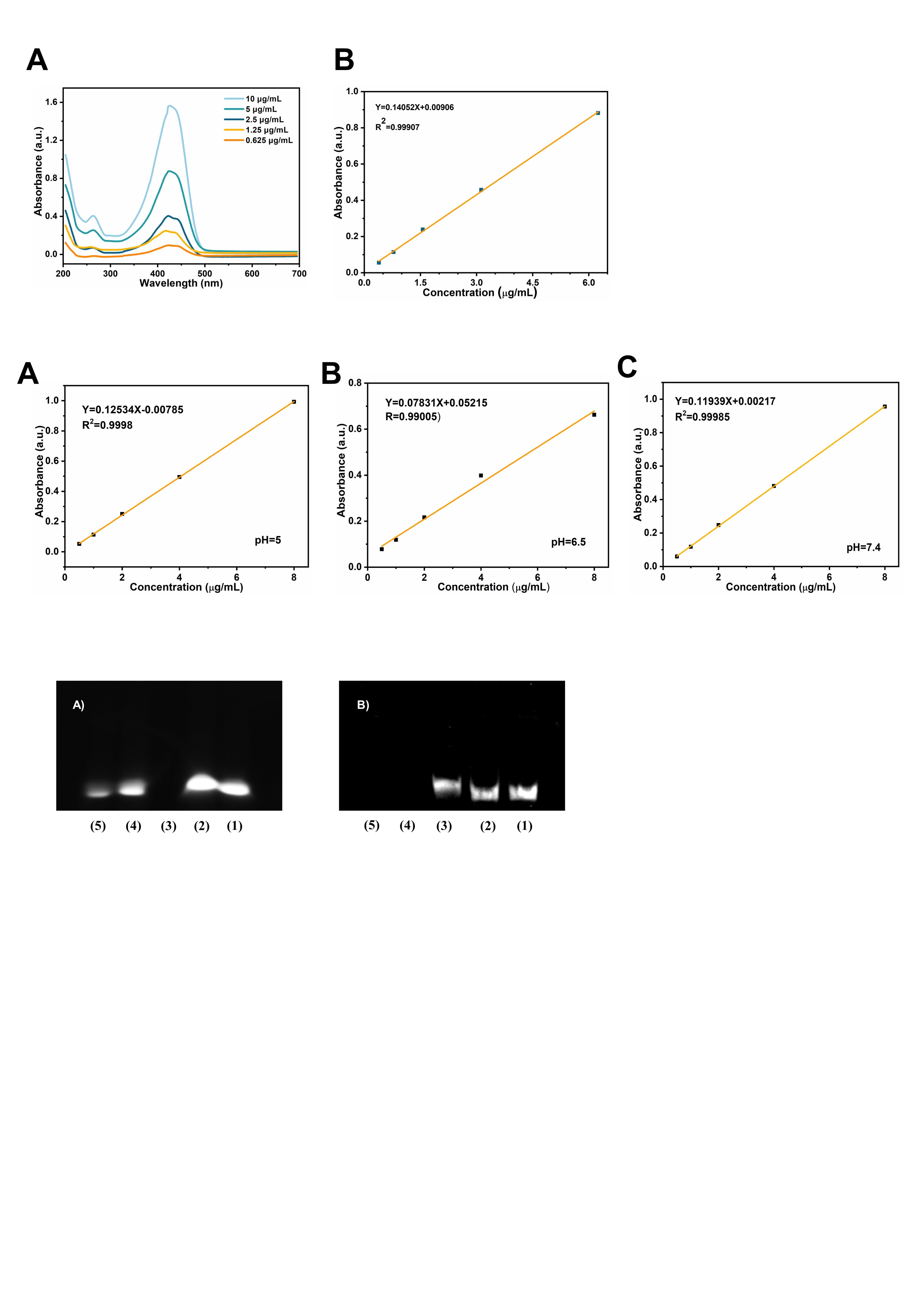


**Figure S4. A)** Agarose gel electrophoresis of (1) naked siRNA, (2) MCS NP_S_ (DLC of MC = 25.9%), (3) supernatant of MCS NPs (DLC of MC = 25.9%, (4) MCS NPs (DLC of MC = 42.5%), and (5) supernatant of MCS NPs (DLC of MC = 42.5%). **B)** Agarose gel electrophoresis of (1) naked siRNA, (2) MS NPs, (3) MCS NPs, (4) the supernatant of MS NPs, and (5) the supernatant of MCS NPs.


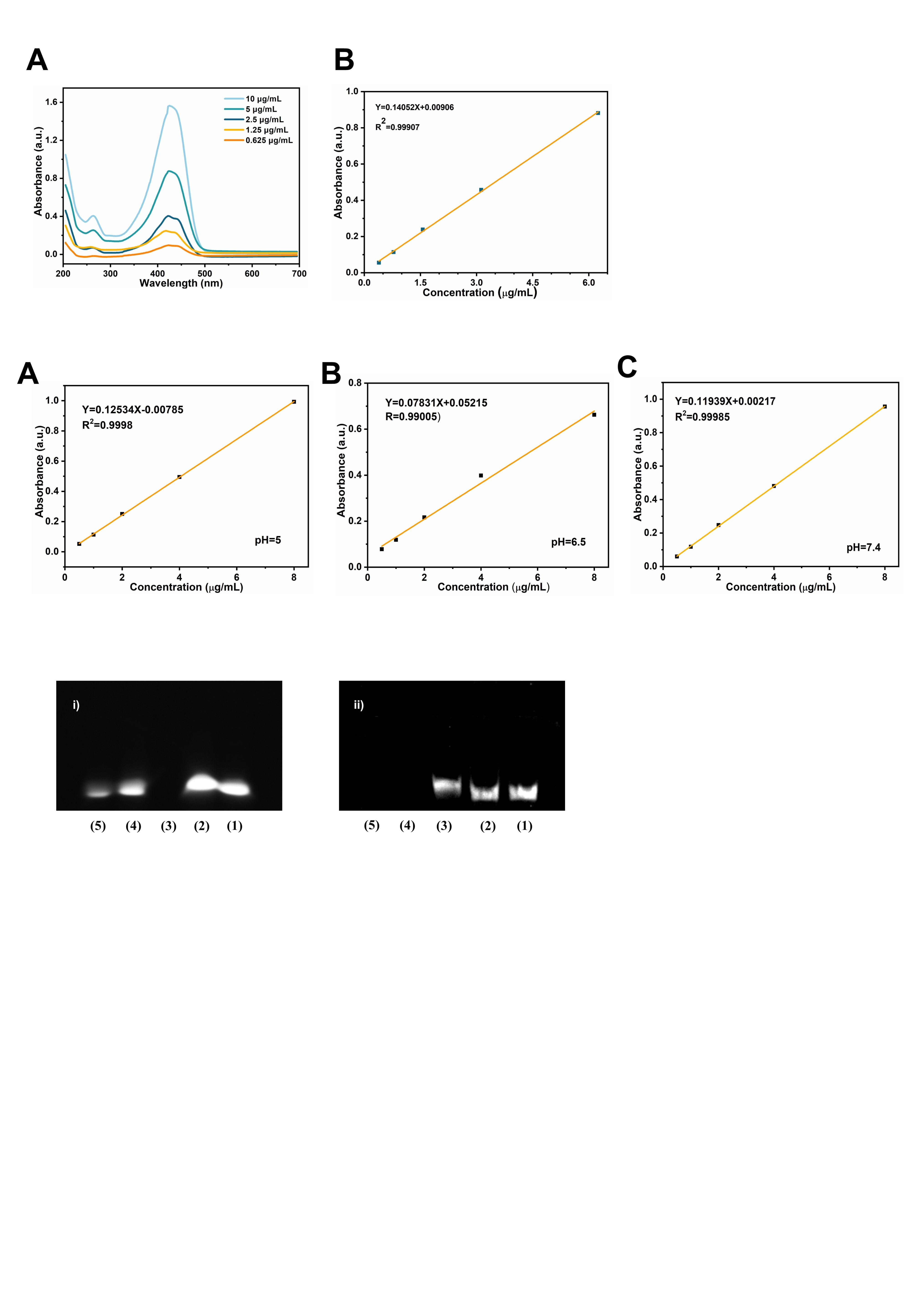


**Figure S5.** The standard curve line of CCM in PBS-Tween 80 with **A)** pH = 5.0, **B)** pH = 6.5, and **C)** pH = 7.4.

**
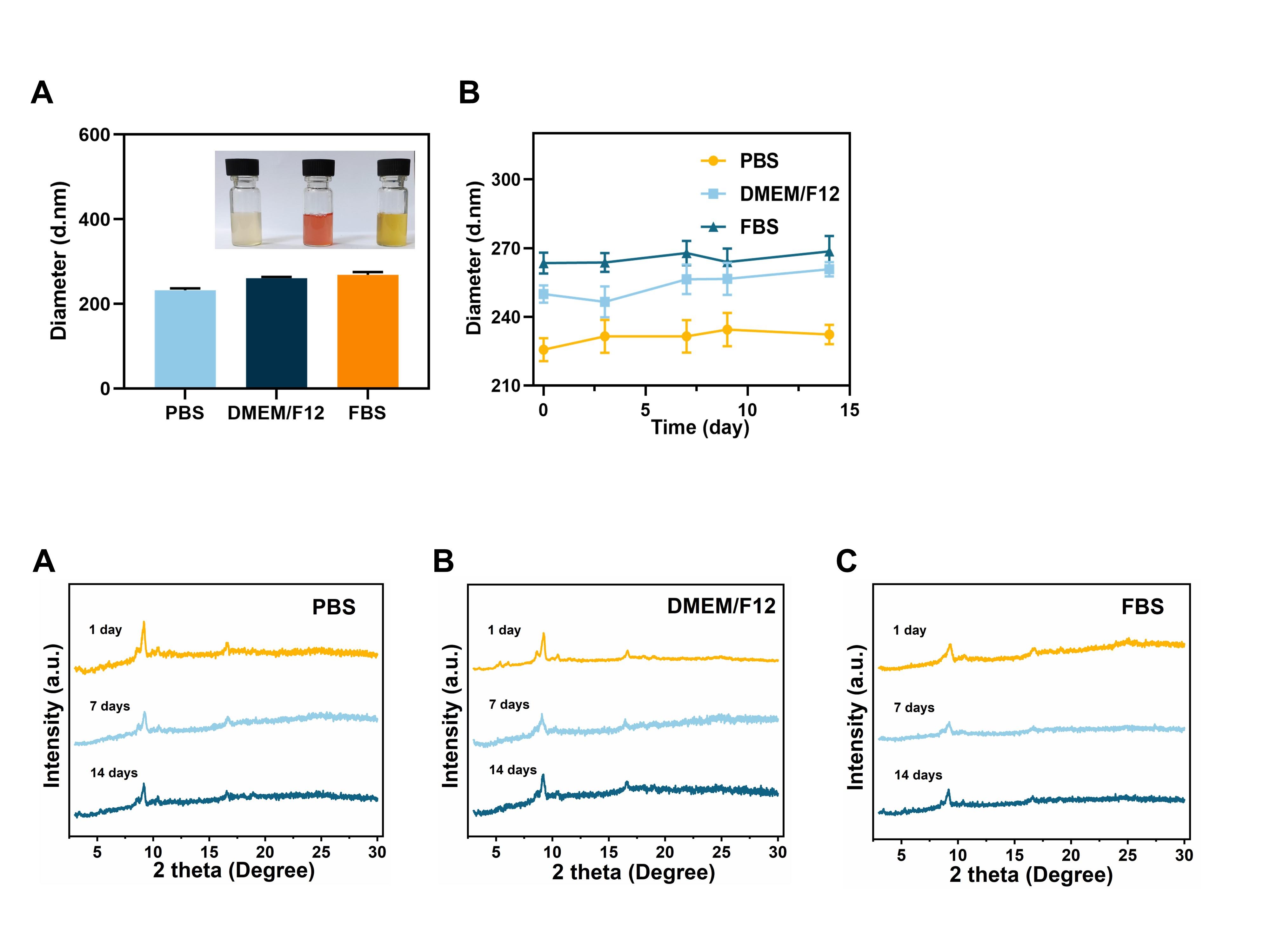
**

**Figure S6. A)** Hydrodynamic diameters of MCS NPs after being incubated in different physiological solutions (PBS, DMEM/F12, FBS) for 14 days. **B)** The time-dependent DLS sizes of MCS NPs in various solution within 14 days.


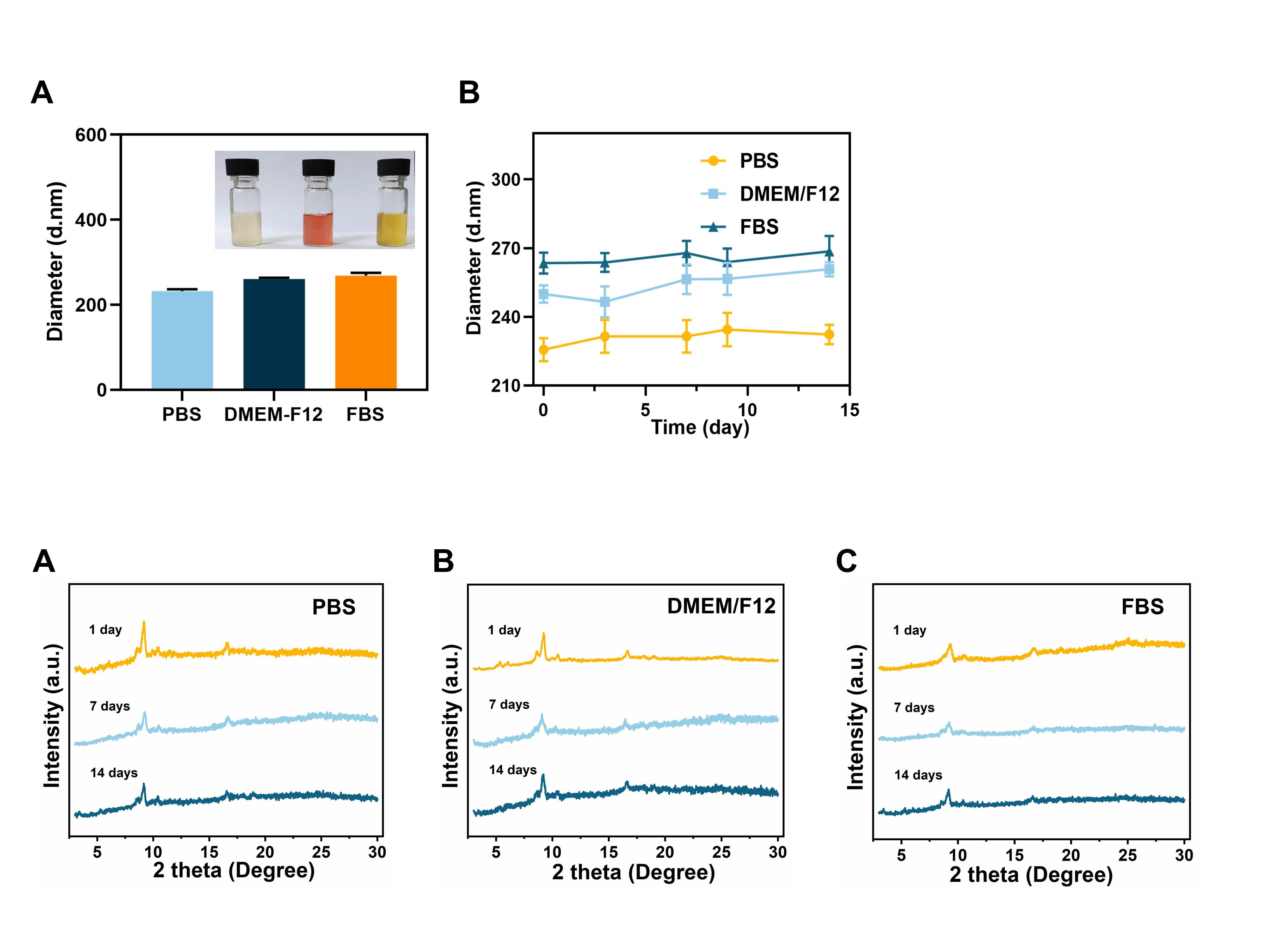


**Figure S7.** The time-dependent PXRD patterns of MCS NPs after being incubated in **A)** PBS **B)** DMEM/F12 and **C)** FBS for 14 days.


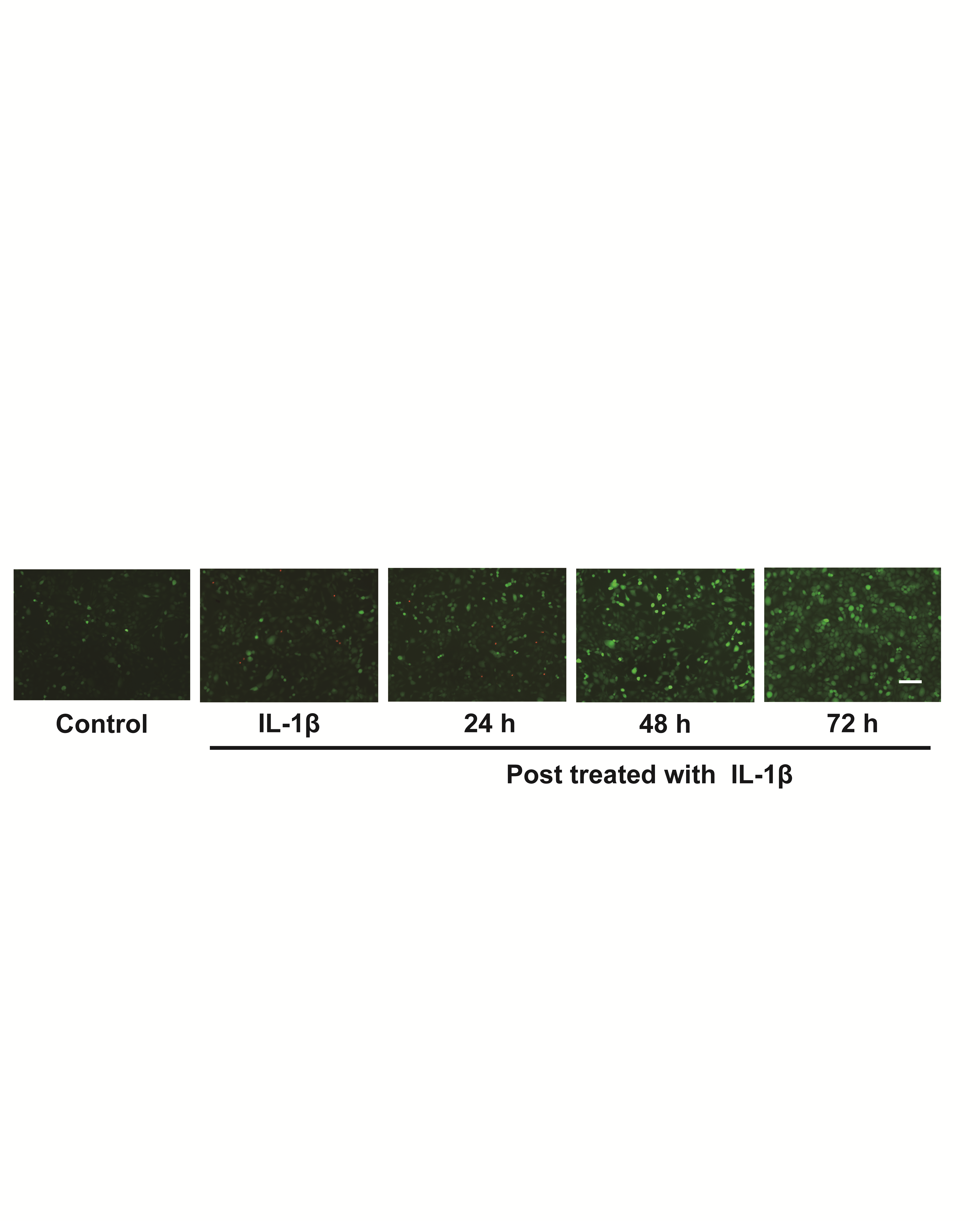


**Figure S8.** Live/dead assay of IL-1β-stimulated chondrocytes after treating with MCS NPs for 24, 48, and 72 h. (Scale bar: 200 μm)


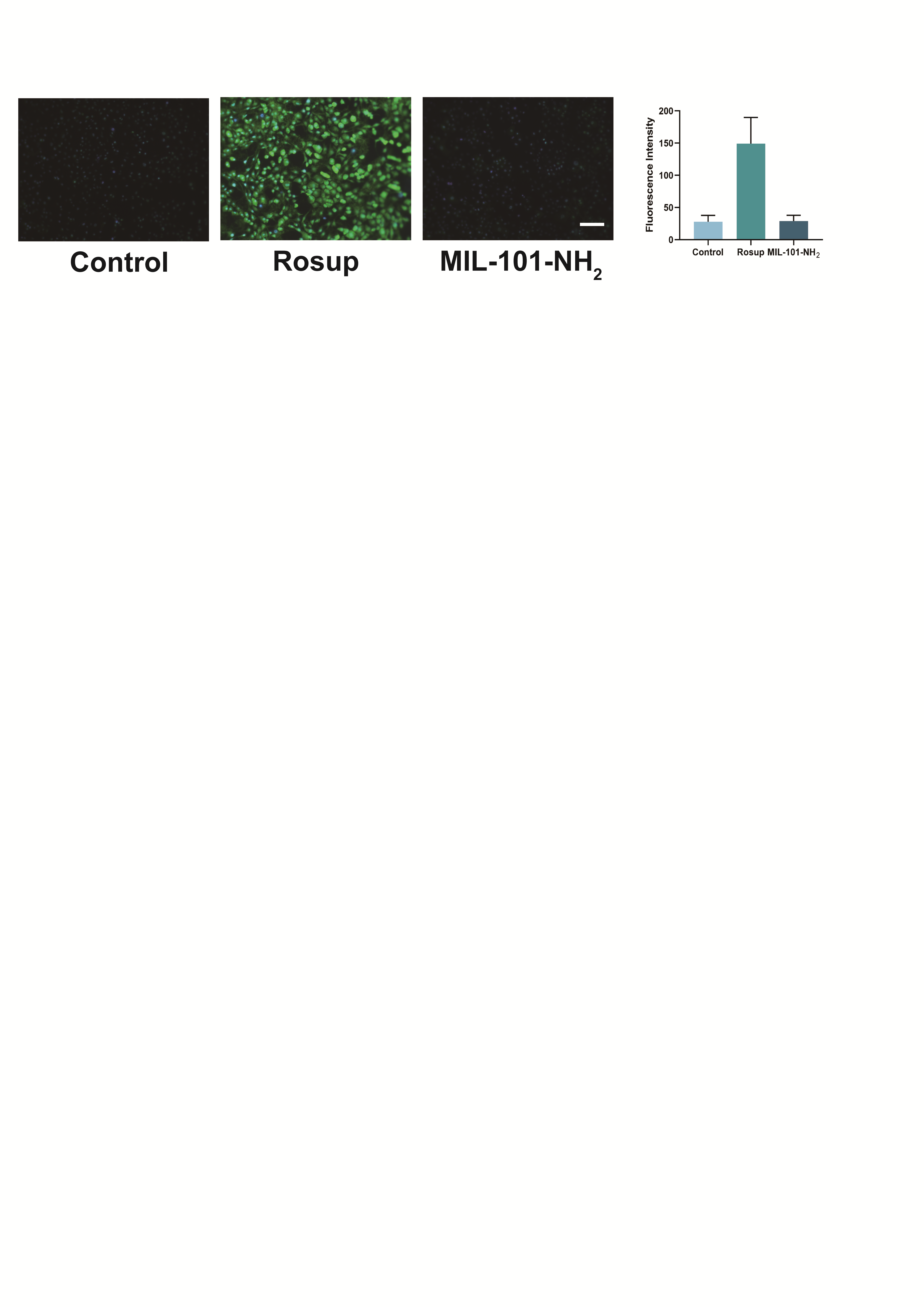


**Figure S9.** ROS production induced by MIL-101-NH_2_ after incubation in chondrocytes for 24 h. (Scale bar: 200 μm) Data was presented as the mean ± SD (n=3).


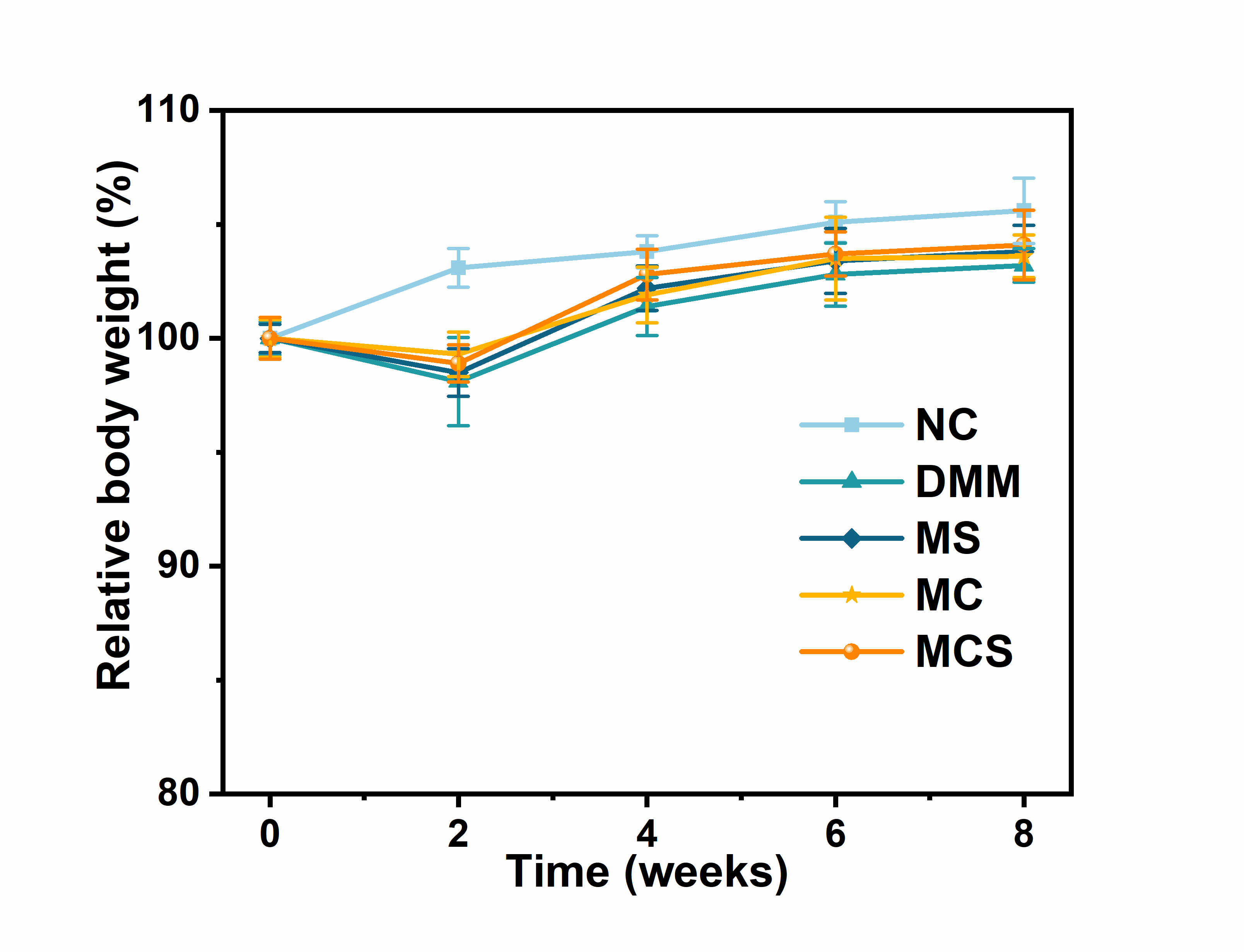


**Figure S10.** Relative body weight change of mice in 8 weeks.

**Table S1.** The DLCs and DLEs of different weight ratios of MIL-101-NH_2_ and CCM.

| Mass ratio of MIL-101-NH_2_ and CCM | DLC(%) | DLE(%) |
| --- | --- | --- |
| 2:1 | 30.8% | 42.3% |
| 1:1 | 25.9% | 69.5% |
| 1:2 | 33.7% | 56.9% |
| 1:3 | 42.5% | 51.4% |

**Table S2.** Different siRNA and objectives used in the experiments.

| **siRNA** | **Objectives** | **Experiments** |
| --- | --- | --- |
| siHIF-2 | Complexation of MIL-101@CCM-siRNA | DLS, zeta potential, UV-vis, and agarose gel electrophoresis |
|  | *In vitro* cytotoxicity | MTT |
|  | HIF-2α silencing | qRT-PCR and *in vivo* OA treatment |
| siCy5 | Loading efficiency and release profile | Fluorescence spectrum |
|  | Cellular uptake and lysosome escape | CLSM |

**Table S3**. DLS and zeta potential of MIL-101-NH_2_@CCM-siRNA_X_ complexes at different weight ratios.

|  |  |  | **MIL-101@CCM-siRNA_5_** | **MIL-101@CCM-siRNA_10_** | **MIL-101@CCM-siRNA_15_** | **MIL-101@CCM-siRNA_20_** | **MIL-101@CCM-siRNA_25_** | **MIL-101@CCM-siRNA_30_** | **MIL-101@CCM-siRNA_35_** |
| --- | --- | --- | --- | --- | --- | --- | --- | --- | --- |
| **w/w^a)^** |  |  | 5:1 | 10:1 | 15:1 | 20:1 | 25:1 | 30:1 | 35:1 |
| **Size (nm)** |  |  | 237.6±9.6 | 235.5±7.7 | 239.5±7.4 | 230.7±8.5 | 232±9.3 | 225.9±5.4 | 226.8±7.2 |
| **ζ(mV)^b)^** |  |  | -2.9±3.53 | -10.3±3.40 | -14.5±4.64 | -17.8±3.78 | -19.6±3.12 | -20.7±2.34 | -20.9±3.67 |

a) w/w: weight ratios of MIL-101-NH_2_@CCM to siRNA

b): zeta potential

***Drug loaded data:***


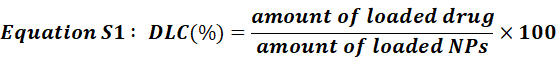


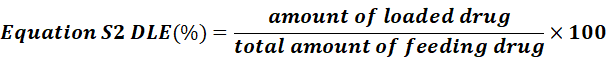


## Equation S1 and S2. Formulas to calculate loaded CCM (DLC and DLE) in MIL101-NH_2_

***Drug release data:***


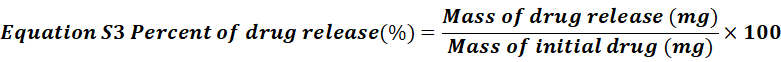


**Equation S3.** Formula to calculate percent of CCM and siRNA release.
